# An innovative approach to characterizing the refractive indices and effective densities of internally mixed light-absorbing aerosol particles

**DOI:** 10.1080/02786826.2025.2468966

**Published:** 2025-03-06

**Authors:** Gwen R. Lawson, Simon Xi Chen, Guy Collins, Naomi Lawson, Kate Szpek, James Bowles, James Allan, Justin M. Langridge, Michael I. Cotterell

**Affiliations:** a School of Chemistry, University of Bristol, Bristol, United Kingdom; b Met Office, Exeter, United Kingdom; c Department of Earth and Environmental Sciences, University of Manchester, Manchester, United Kingdom; d Department of Chemistry, University of Oxford, Oxford, United Kingdom

**Keywords:** Hans Moosmüller

## Abstract

To improve our understanding of how light interacts with internally mixed light absorbing aerosol particles, we establish an integrated measurement platform enabling concurrent determinations of the complex refractive indices (*m* =* n* + *ik*) and effective densities (*ρ*) of aerosol particles. Cavity ring-down and photoacoustic spectroscopy are used to measure the extinction and absorption cross-sections, respectively, for aerosol particles classified by their aerodynamic size using the Cambustion Aerodynamic Aerosol Classifier. We report measurements on laboratory generated aerosol particles composed of ammonium sulfate (non-absorbing inorganic), sucrose (non-absorbing organic), nigrosin (strong light-absorbing organic), and two-component particles comprised of internal mixtures of nigrosin with each non-absorbing species. The accuracy and precision of measured cross-sections and retrieved *m* are assessed, and we demonstrate improved precision in these quantities retrieved for aerodynamically classified particles compared to approaches that utilize mobility classification. We show that accurate knowledge of the variations in *ρ* with mixture composition are essential for predicting *m* for internally mixed particles using mixing rules. For organic mixtures of sucrose and nigrosin, *n* and *k* are predicted accurately by mass fraction weightings of pure component values, and ideal mixing between components is observed. For organic-inorganic mixtures of nigrosin with ammonium sulfate, *n* varies non-linearly with composition and cannot be predicted by linear mixing rules. Instead, a mole fraction weighting of molar refraction, incorporating changes in particle mass density on mixing, is needed. These evaluations of refractive index models provide useful insights for researchers developing atmospheric models or inferring particle physicochemical properties from optical spectroscopy data.

## Graphical abstract

**Figure:**
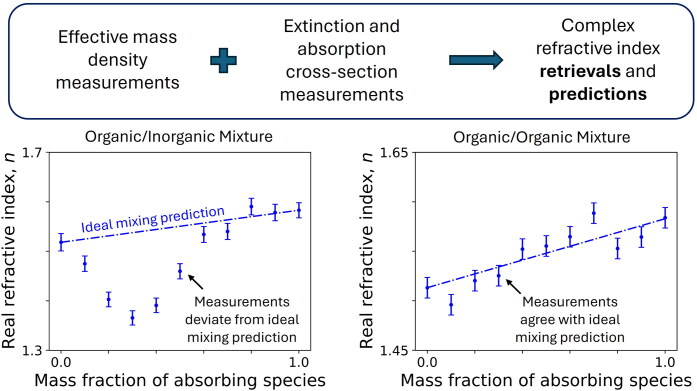

## 1. Introduction

The refractive index (RI, *m*) is a microphysical property of a substance related to the mass density, molecular weight and mean molecular polarizability. It is a complex valued quantity, with *m = n + ik*, in which *n* and *k* are the real and imaginary components, respectively (Liu and Daum 2008). The real component represents the ratio of the speed of light in vacuum to its phase speed in the substance of interest, while the imaginary component is the intensive property characterizing the attenuation of light (i.e., absorption) by the substance. Both *n* and *k* depend on the wavelength of light. For spherical and homogeneous aerosol particles with a known size, *m* can be input to the Lorenz-Mie equations to calculate important optical properties that govern aerosol-light interactions.

Aerosols have an overall cooling effect on the surface temperatures of Earth, but uncertainties exist surrounding the exact contributions of the different interactions they have with light (IPCC 2021). These uncertainties are particularly large for light absorbing particles. Indeed, the description of aerosols represents one of the largest uncertainties in climate models and improvements in the understanding of their optical properties are needed. The interactions of aerosol particles with light are characterized by their optical cross-sections. The extinction cross-section ($\sigma_{\text{ext}}$) quantifies the ratio of the power removed from an incident beam of light by an aerosol particle to the total incident irradiance, and is the sum of the scattering ($\sigma_{\text{sca}})$ and absorption ($\sigma_{\text{abs}})$ cross-section:

(1) $$\sigma_{\text{ext }}=\sigma_{\text{sca }}+\sigma_{\text{abs }}$$

The magnitudes of $\sigma_{\text{sca}}$ and $\sigma_{\text{abs }}$ depend critically on the particle size, morphology and complex refractive index and need to be quantified accurately to determine the single scattering albedo that is input into radiative forcing calculations (Haywood and Shine 1995). Measurements of one or a combination of cross-sections for particles of a known size distribution allow the complex refractive index to be characterized through an inverse retrieval method. This work exploits this retrieval approach to determine *n* and *k* for internally mixed aerosol particles with varying degrees of light absorption.

The compositions, and therefore refractive indices, of aerosol particles can differ considerably from those of bulk macroscale materials, necessitating direct aerosol measurements. Previous studies of aerosol plumes (or *ensembles*) that explored refractive indices for aerosol particles usually involved characterizing the extinction, scattering or absorption coefficients (*α*) of size-selected aerosol particles in combination with the particle number concentration (*N*), allowing calculation of the corresponding ensemble-mean optical cross-sections (*σ = α/N*). Values for *n* and *k* are then retrieved by comparing these measured cross-sections to model predictions from Lorenz-Mie theory (Bohren and Huffman 1998). Several studies have used an extinction-only method for the retrieval of complex RI values, commonly using cavity ring-down spectroscopy (CRDS), which is an accurate technique for measuring the extinction coefficients of aerosols (Lang-Yona et al. 2010; Michel Flores et al. 2012; Trainic et al. 2012; Zarzana et al. 2012; Toole, Renbaum-Wolff, and Smith 2013; Washenfelder et al. 2013). CRDS is a highly sensitive technique owing to the long optical pathlength achieved by reflecting a laser beam several thousand times through the aerosol-laden sample within an optical cavity.

Most of the aforementioned studies have focused on non-absorbing aerosol particles and measured only the ensemble-mean extinction cross-sections. However, it is imperative that studies of aerosol optical properties and their connection to the physicochemical properties of the constituent particles are extended to light-absorbing particles. Indeed, retrievals of complex refractive indices from extinction-only measurements are subject to higher levels of uncertainty and it is important to measure concurrently a further cross-section (such as that for absorption) to reduce these uncertainties. Zarzana, Cappa, and Tolbert (2014) compared RI retrievals from simulated extinction cross-sections with those that also included absorption cross-sections and showed that the addition of the absorption cross-sections greatly improved the retrieval accuracy for both absorbing and purely scattering species. Photoacoustic spectroscopy (PAS) has become a favored non-intrusive technique for measuring *in situ* absorption coefficients directly for aerosol-laden samples. PAS relies on the photoacoustic effect (Haisch 2012): a sample that absorbs light from an intensity-modulated laser beam liberates its energy by the transfer of heat to the surrounding bath gas that subsequently expands rapidly, thereby generating a pressure (acoustic) wave. PAS has been shown to yield accurate aerosol absorption coefficient measurements under dry conditions for sub-µm particles (Lack et al. 2006). The accuracy of these measurements depends critically on the calibration of the photoacoustic response to an absorption coefficient; calibrations utilizing ozone-laden gas samples are robust (Davies et al. 2018) and are used in our work. Absorption measurements using PAS combined with extinction measurements from CRDS has been shown to be a good approach for laboratory measurements of aerosol optical properties (Lack et al. 2006; Lambe et al. 2013; Nakayama et al. 2013; Radney and Zangmeister 2018; Cotterell et al. 2020) and are therefore used in this work.

Cotterell et al. (2020) used CRDS and PAS to measure extinction and absorption cross-sections and then retrieve the complex RI for aerosol particles comprised of nigrosin (a light-absorbing organic dye), ammonium sulfate (a non-absorbing inorganic salt), and four different mixing ratios of these two species. A differential mobility analyzer (DMA) was used to select aerosol particles by their electrical mobility diameter prior to CRDS and PAS measurements of their extinction and absorption coefficients respectively. However, selection of particles using a DMA leads to large uncertainties in the particle size distribution that degrades the accuracy in RI retrievals. Principally, these uncertainties arise from those in the (Boltzmann-like) charge distribution imparted by a bipolar neutralizer to the polydisperse aerosol ensemble prior to mobility classification. This charging process results in multiple charge artifacts; most of the selected particles will be singly charged with the desired mobility diameter, but a non-negligible fraction will have multiple charges and will be larger than the target mobility diameter (Miles et al. 2011). These multiply charged particles must be accounted for in the retrieval of refractive indices, but a lack of accurate characterization of the charge distribution and how this depends on flow conditions introduces systematic errors in the retrieved *n* and *k*. Cotterell et al. (2020) reported that typical uncertainties in the aerosol particle charge distribution result in systematic errors in the retrieved RI of 3.6% for *n* and 8.5% for *k*. Scattering cross-section measurements using a dual DMA system by Khalizov et al. (2009) gave values 17% to 47% higher than values calculated from Lorenz-Mie theory for ammonium sulfate aerosol particles as a result of multiply charged particles. Similarly, for polystyrene nanospheres the authors reported cross-sections 2% to 12% higher than expected and a larger error at smaller particle sizes was observed. Many studies have not accounted for these multiply charged particles or instead used methods to minimize the fraction of particles with multiple charges. However, these methods reduce particle number concentrations and consequently degrade the sensitivity of downstream optical measurements.

Size selection can instead be achieved without charging aerosol particles by using an aerodynamic aerosol classifier (AAC), developed by Tavakoli and Olfert (2013). The AAC is a recent innovation that selects aerosol particles based on their relaxation time (Yao et al. 2020), which is the characteristic time of approach to constant velocity when subjected to both centrifugal and drag forces and can be expressed in terms of an aerodynamic diameter. In addition, the AAC has a much greater transmission efficiency than a DMA; Johnson et al. (2018) found this efficiency to be up to five times higher. This improved transmission efficiency results in higher particle number concentrations, allowing for measurements of optical attenuation (e.g., extinction and absorption) coefficients with improved sensitivity. In this work, in contrast to previous studies, we use an AAC to select particles from a polydisperse ensemble by their aerodynamic size. Extinction and absorption coefficients are then measured using CRDS and PAS instruments, respectively. Our optical spectroscopy instruments were used previously to sample AAC-selected particles during the Soot Aerodynamic Size Selection for Optical properties (SASSO) measurement campaign, which characterized the optical properties of soot particles generated from controlled combustion of different sources (Hu et al. 2021, 2023). However, a complete assessment of the accuracy and precision of complex RI retrievals of AAC selected particles is not yet reported. We provide a thorough assessment of the uncertainties in retrievals of *n* and *k* from CRDS and PAS measurements of extinction and absorption cross-sections, respectively, for aerodynamically classified particles at optical wavelengths of 405 nm and 658 nm.

In this work, we examine the optical properties of two-component internally mixed light-absorbing particles. We assess the ability of several optical mixing models to reproduce the measured variations of *n* and *k* with particle composition. Previous work to explore the optical properties of internally mixed light absorbing aerosol particles is limited. Cotterell et al. (2020) explored particles comprised of two-component internal mixtures of an inorganic (ammonium sulfate) and organic (nigrosin) species in controlled mass ratios. The retrieved values for *n* of mixtures showed considerable deviations from linear mass or volume fraction weightings of RI of pure components, while a physically based model that accounted for variations in the mixture mass density with composition successfully reproduced the retrieved *n*. The authors used a mass density parameterization for the mixtures reported by Radney and Zangmeister (2018) and did not characterize directly the densities of the particles formed from their aerosol generation and conditioning set-up. This previous work provided measurements at only four different mass ratios that limited a rigorous assessment of the performance of optical models for mixtures. Here, we report values for *n* and *k* over a comprehensive range of mixture compositions, contrasting the mixing behavior of an inorganic-organic mixture (ammonium sulfate and nigrosin) with an organic-organic mixture (sucrose and nigrosin). These measurements are used to develop and test different mixing rules for predicting *n* and *k*. Moreover, our measurement framework allows concurrent determinations of the particle densities, which are required for application of physically based refractive index mixing models.

## 2. Experimental methods

Sections 2.1–2.3 describe the experimental methods used to measure the ensemble-mean extinction and absorption cross-sections and mobility size distributions for aerodynamically classified aerosol samples. The method for retrieval of *n* and *k* from these measurements is provided in Section 2.4. Section 2.5 describes how the particle mass density was determined, and Section 2.6 describes how particles were collected and imaged using scanning electron microscopy.

### 2.1. Aerosol generation, size selection and mobility size measurements

**Figure 1.**
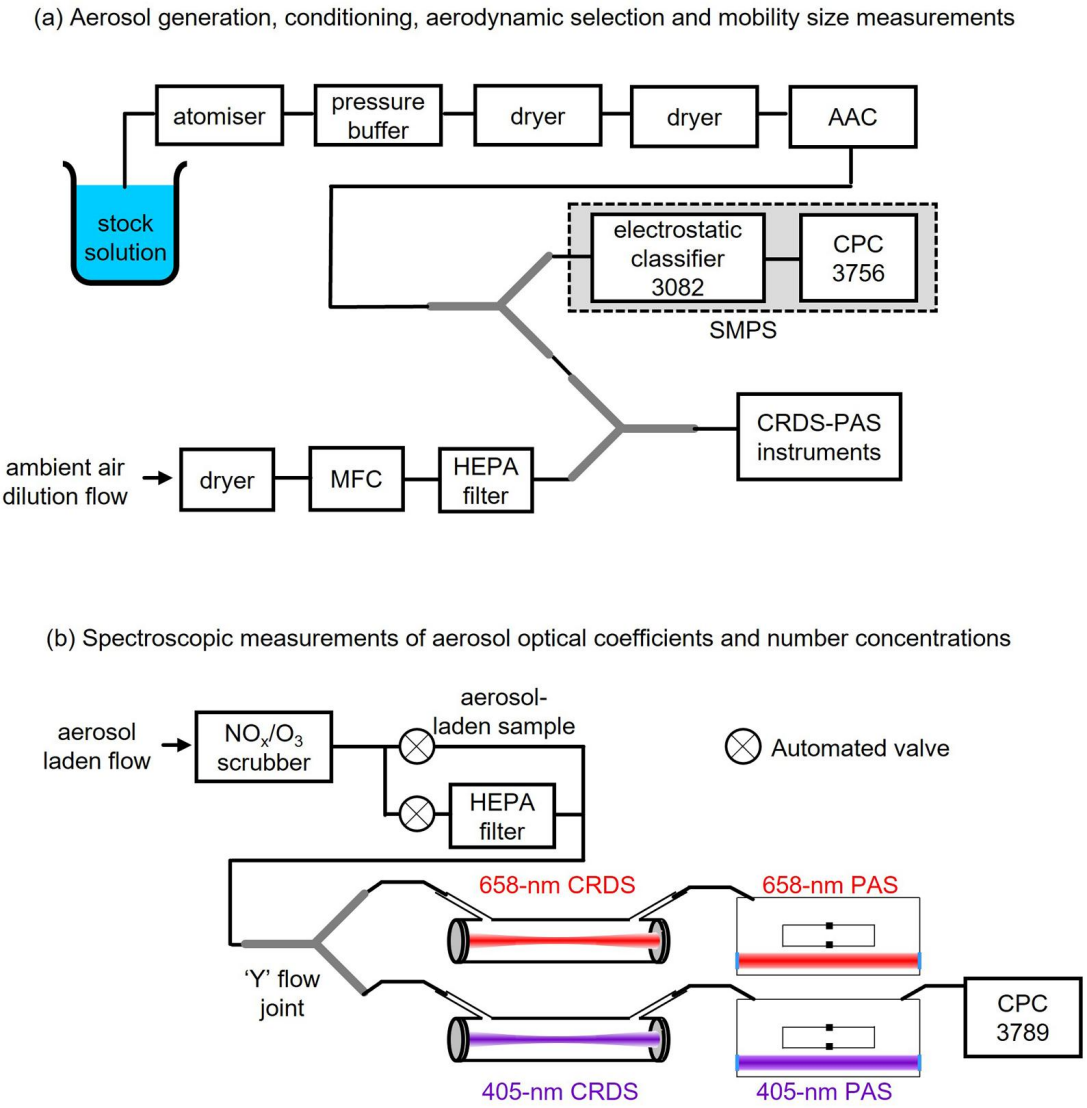
(a) The experimental configuration for aerosol generation, conditioning, aerodynamic size selection, and mobility size distribution measurements prior to dilution of the flow for optical characterization in CRDS-PAS instruments. (b) The configuration for spectroscopic measurements of aerosol extinction and absorption coefficients by CRDS and PAS, respectively. A CPC sampled from the exhaust of the 405-nm PAS cell to provide aerosol number concentration measurements.

Figure 1a shows the experimental configuration used to generate, dry, and aerodynamically classify aerosol samples for downstream characterization. Aqueous stock solutions with a total solute concentration of 4 g L^−1^ were prepared through dissolution of the desired solute or mixture of solutes in deionized water (Purite HP; purity > 10 MΩ cm). The solutes used were ammonium sulfate (Sigma-Aldrich, CAS number 7783-20-2), water-soluble nigrosin dye (Sigma-Aldrich, CAS number 8005-03-6, lot number MKCC1553), and sucrose (Sigma-Aldrich, CAS number 57-50-1, lot number BCBM5122V). The solutions were atomized using HEPA-filtered compressed air from a portable test aerosol generator (TSI, Model 3073) with a nozzle pressure of 600 hPa, before they were passed into a pressure buffer, consisting of a glass vessel with one output port vented to ambient air *via* a HEPA filter, to maintain downstream pressure. The aerosol sample was then dried by passing it through two silica gel diffusion driers (Aerosol Diffusion Drier, Cambustion).

The dry aerosol-laden flow was drawn through an AAC (Cambustion), which selected aerosol particles based on their relaxation time, which is directly related to the aerodynamic diameter (*d*_ae_; the diameter of a unit density spherical particle with an equivalent terminal settling velocity) (Tavakoli and Olfert 2013). Aerodynamic diameters were selected between 100 nm and 400 nm in 25 nm intervals. The resolution parameter (*R*_s_) of the AAC, which is the ratio of the set aerodynamic diameter to the full width at half maximum of the transfer function, was set to 20. The flow rate through the AAC, which was governed by the downstream instruments that drew the aerosol-laden air through the AAC, was ∼0.9 L min^−1^.

After size selection by the AAC, the aerosol flow was divided using a ‘Y’ flow splitter. A scanning mobility particle sizer (SMPS) sampled from one output; the SMPS consisted of an electrostatic classifier (TSI 3082) and an ultrafine condensation particle counter (CPC, TSI 3756). On entering the SMPS, the sample was drawn through an internal impactor with an orifice diameter of 710 µm (and *d*_50_ cutoff diameter of ∼1.33 µm depending on the precise flow rate) to the electrostatic classifier. A bipolar charge distribution was imparted on the aerosol ensemble using a soft x-ray neutralizer (TSI 3088), and this charged aerosol population then passed to a DMA (TSI 3081). The SMPS was used to measure electrical mobility size distributions of the aerodynamically classified aerosol sample continuously, with a scan time of 90 s and a purge time of 15 s, across the mobility diameter range of (12.52 to 537.61) nm using a sample flow (governed by the flow rate setting of the CPC) of 0.3 L min^−1^. The sheath flow was set to 4 L min^−1^, which ensured a ratio of sample-to-sheath flow of at least 1:10 as recommended by the manufacturer. Corrections for multiple charging artifacts and diffusion losses were applied by the manufacturer’s software. Mobility size distributions were used to calculate predicted optical cross-sections as discussed in Section 2.4 and for calculations of effective particle mass density as described in Section 2.5. The aerosol sample flow from the second output of the splitter was diluted using dried and HEPA-filtered laboratory air, the flow of which was maintained at 0.5 L min^−1^ using a mass flow controller (MFC, Alicat) before it was drawn into the CRDS-PAS instrument for optical coefficient measurements.

Calibrations of the AAC and SMPS were performed using polystyrene spheres (PSS) (see Section S1 of the online supplementary information [SI]). The existing calibration of the SMPS was validated using the PSS, and no further correction was required. From the AAC calibrations, a correction coefficient was determined to account for a small difference between the user-selected and measured aerodynamic diameters. This average multiplicative factor was determined to be 0.954 ± 0.011 and was used to correct the selected aerodynamic diameter during the data analysis process.

### 2.2. Extinction and absorption coefficient measurements

The CRDS and PAS spectrometers used to measure extinction and absorption coefficients were contained within an instrument developed by the Met Office, used previously for field (Davies et al. 2019; Peers et al. 2019, 2021) and laboratory measurements (Davies et al. 2018; Cotterell et al. 2020, 2019a) of aerosol optical properties. The instrument consists of four CRDS and five PAS spectrometers which operate at optical wavelengths of 405, 514 and 658 nm. This work only utilized the 405 nm and 658 nm wavelength spectrometers. Thorough descriptions of the instrument can be found in previous work (Davies et al. 2018; Cotterell et al. 2019b). The selection of spectrometers used, and their configuration is shown in Figure 1b. Aerosol samples first passed through an activated carbon honeycomb scrubber (Mast Carbon) to remove any trace NO_x_ or O_3_ gas, which absorb light in the visible spectrum and would bias measurements. The sample flow was then split between a 405-nm and 658-nm sampling line in parallel and passed through the CRDS spectrometer prior to the PAS spectrometer in a serial flow configuration. MFCs were used to maintain a flow rate of 0.6 L min^−1^ through each sampling line. We refer the reader to Sections S2 and S3 of the online SI for a thorough description of our CRDS and PAS spectrometers and their operating principles. For each selected aerodynamic diameter, the extinction and absorption attenuation coefficients were recorded at a 1 Hz sampling frequency for 5 min. Prior to measurements for a given aerodynamic diameter, the aerosol sample was passed through a high efficiency particulate air (HEPA) filter upstream of the spectrometers for 2 min to remove aerosol particles and provide a background measurement of the cavity ring-down time and the background noise in the photoacoustic signal. Additionally, for the PAS spectrometer, a speaker built into the spectrometer was used to excite the resonant eigenmode of the PAS cell to measure its resonance frequency and quality factor (see Sections S3 of the SI). These quantities are required to correct the measured photoacoustic signal (prior to application of a calibration to convert to absorption coefficient) for any changes in the acoustic properties of the PAS cell that may arise from changes in pressure and bath gas composition.

### 2.3. Aerosol number concentration measurements

Conversion of the attenuation coefficients of aerosol samples measured directly by the CRDS-PAS instrument to their corresponding ensemble-mean single particle optical cross-sections relied on measurements of total particle number concentration. The relationship between the optical coefficient (α), cross-section (σ) and number concentration (N) is:

(2) α=Nσ

A CPC (TSI 3789), operating with a flow rate of 0.6 L min^−1^ and sampling rate of 1 Hz, was used to measure the particle number concentration exiting the 405-nm PAS cell. The measured extinction and absorption coefficients were divided by the corresponding aerosol number concentration to calculate cross-sections. These cross-sections were calculated at the sampling rate of 1 Hz for each 5-min measurement, before the mean value over this 5-min period was calculated for each value of selected aerodynamic diameter. The standard deviations in the calculated cross-sections were determined through an error propagation of the standard deviations in the measured mean optical coefficients and number concentrations. The difference in transmission of aerosol particles between the two different sampling lines was characterized by measuring a multiplicative transmission factor (*T*_658_) that was used to correct the number concentration measured by the CPC at the exhaust of the 405-nm PAS cell to a value relevant to that at the 658-nm PAS cell. This transmission factor was calculated to be *T*_658_ = 1.243 ± 0.019 (see Section S4 of the SI). This ∼24% difference in transmission between the sampling lines is larger than would be expected from differences in the splitting from the ‘Y’ flow joint alone. The differences in purge flow dilution between the two sampling lines for this flow configuration likely contributes to this large discrepancy. The flow configuration and sample flow rates used in this work differ to those used previously with the same instrument and this larger difference in flow transmission is characteristic of these conditions. The total mirror purge flow of 0.1 L min^−1^ is split passively by a purge manifold to the four mirrors of the two CRDS spectrometers and is not regulated actively using a mass flow controller. Each of the four outputs from this manifold use a critical orifice (O’Keefe, part number IL-H0132-4, orifice diameter 0.10 mm) to regulate passively the purge flow between the four mirrors. Therefore, differences between purge flows in the two sampling lines is likely a large contributor to this higher-than-expected difference in transmission. While the instrument was pressure tested, and we tried to minimize differences in losses to the sampling line by using conductive tubing, minimizing bends in the tubing and having approximately equal sample line lengths, it is possible that small leaks and static charge on surfaces provided additional particle loss sources in one of the sampling lines.

### 2.4. Complex refractive index retrievals

Measurements of extinction and absorption cross-sections for discrete values of *d*_ae_ over the range 100 nm to 400 nm in 25 nm intervals facilitated the retrieval of the effective RI of the particles. Complex refractive indices were retrieved by performing a grid search starting within an expected range of *n* and *k*, followed by further grid searches with increasingly smaller step sizes until the step sizes in *n* and *k* were less than 0.001 and 0.0001, respectively. For each pair of trial values for *n* and *k*, Lorenz-Mie theory was used to calculate model values for the ensemble-mean single particle extinction and absorption cross-sections ($\sigma_{\text{model},\text{ext},i}$ and $\sigma_{\text{model},\text{abs},i,}$ respectively) using the mobility size distribution data measured by the SMPS. The mobility size distributions often exhibited multiple (typically, two or three) peaks. Section S5 of the SI shows examples of these peaks. The origin of these multiple peaks is attributed to multiply charged particles, since the charge applied to the sample by the x-ray neutralizer was Boltzmann-like. Importantly, because the aerosol samples had a monomodal particle size distribution owing to the initial size selection by the AAC, the dominant peak in the measured mobility size distribution corresponded to singly charged particles and peaks appearing at smaller particle diameters were artifacts corresponding to particles with higher charge states. These multiple peaks in mobility diameter measurements for AAC-selected aerosol samples were examined by Vokes et al. (2022) and the authors show the presence can be attributed to multiply charged particles. Therefore, these multiply charged particle artifacts were not included in calculations of cross-sections. As a result, only the singly charged (*q* = 1) peak with the largest amplitude was considered in calculations, as this corresponded to the true mobility size distribution of the particles for a given *d*_ae_. To extract the size distribution for this peak, a bimodal lognormal distribution was fit to the full measured mobility size distribution using the same approach reported by Vokes et al. (2022), and the median mobility diameter and geometric standard deviation characterizing the distribution of the singly charged particles were determined. A bimodal fit was chosen as it improved the fit to the data and better captured the *q* = 1 peak compared to a monomodal fit. It was only important to fit accurately the data corresponding to the singly charged peak, and therefore lognormal distributions of higher orders were not required. To calculate the model optical cross-sections, the determined singly charged mobility distribution was first normalized (such that the integrated particle number concentration was unity). The cross-section for each diameter within this normalized singly charged mobility distribution was calculated by inputting this diameter and trial values for *n* and *k* into the Lorenz-Mie equations, and this cross-section was then multiplied by the corresponding normalized number concentration. In this way, a continuous distribution of a weighted cross-section versus particle mobility diameter was calculated. Finally, a numerical integration (applying the trapezoidal rule) was performed on this distribution to give the ensemble-mean single particle cross-section. Merit functions were then calculated to quantify the level of agreement between experimentally measured ($\sigma_{\text{abs},i}\;\text{and}\;\sigma_{\text{ext},i}$) and modeled ($\sigma_{\text{model},\text{abs},i}\;\text{and}\;\sigma_{\text{model},\text{ext},i}$) cross-sections across all selected aerodynamic diameters. The merit functions for the absorption and extinction cross-sections ($\chi_{\text{abs}}^{2}\;\text{and}\;\chi_{\text{ext}}^{2}$) are defined by:

(3) $$\chi_{\text{abs}}^{2}=\frac{1}{N}\sum_{i}\frac{{\left(\sigma_{\text{abs},i}–\sigma_{\text{model},\text{abs},i}\right)}^{2}}{\epsilon_{\text{abs},i}^{2}}$$

(4) $$\chi_{\text{ext}}^{2}=\frac{1}{N}\sum_{i}\frac{{\left(\sigma_{\text{ext},i}–\sigma_{\text{model},\text{ext},i}\right)}^{2}}{\epsilon_{\text{ext},i}^{2}}\;$$

in which $\epsilon_{\text{abs},i}\;\text{and}\;\epsilon_{\text{ext},i}$ are the standard deviations in the experimental cross-sections, and *N* is the number of selected aerodynamic diameters (*N* = 13). Approximate relative values for standard deviations were 3.8% and 2.5% for the 405-nm extinction and absorption cross-sections respectively, and 3.5% and 3.6% for the 658-nm extinction and absorption cross-sections respectively. To quantify overall agreement of the model and measured cross-sections, $\chi_{\text{abs}}^{2}$ and $\chi_{\text{ext}}^{2}$ were summed ($\chi_{\text{total}}^{2}=\chi_{\text{ext}}^{2}+\chi_{\text{abs}}^{2}$) and the best fit RI corresponded to the pair of {*n*, *k*} refractive index values that minimized $\chi_{\text{total}}^{2}\text{.}$ Applying this $\chi^{2}$ analysis to the simultaneous fitting of extinction and absorption cross-sections to Lorenz-Mie theory is consistent with our work in Cotterell et al. (2020), enabling comparisons of our optical property determinations for aerodynamically classified particles studied in this work with those from mobility classified approaches reported in Cotterell et al. (2020). A similar retrieval algorithm used by Bluvshtein et al. (2012) provided estimates of the confidence region for *n* and *k* directly from these merit functions. Our retrieval algorithm did not provide this information and instead, as described in Section 3, we opted to assess the precision in *n* and *k* from the standard deviation in the retrieved *n* and *k* from three technical replicate measurements of the particle size dependent cross sections, which accounts for the effects of small variations in measurement conditions.

### 2.5. Effective particle mass density measurements

Complex refractive index and mass density have a fundamental connection through the Lorenz-Lorenz relation (Liu and Daum 2008). Studies have previously focused on measurements of material density using techniques such as x-ray crystallography (Johnston and Hutchison 1942; Robie, Bethke, and Beardsley 1967). Effective densities of aerosol particles can differ from these macroscopic material densities and are particularly important for internally mixed particles with multiple components. The effective densities of internally mixed multicomponent systems are often predicted from pure component values using mixing rules. Commonly applied mixing rules, such as those assuming a linear weighting in terms of mass or volume fraction, assume ideal mixing between components. However, Vokes et al. (2022) observed considerable non-ideal behavior when mixing inorganic and organic species, with non-ideal interactions contributing to a volume change on mixing. Effective densities of aerosol particles should be measured directly, rather than estimated by linear mixing rules, where possible.

The mass density of aerosol particles was determined from combined measurements of the average *q* = 1 mobility (*d*_m_) and aerodynamic diameters (*d*_ae_) of aerosol particles, which were obtained from the SMPS scans and the corrected user-specified value of *d*_ae_ input to the AAC. The effective mass density of a sample was calculated using (Vokes et al. 2022):

(5) $$\rho_{e}=\rho_{0}\frac{d_{\text{ae}}^{2}C_{c}(d_{\text{ae}})}{d_{m}^{2}C_{c}(d_{m})}$$

in which *C*_c_ is the Cunningham slip correction factor for either *d*_ae_ or *d*_m_, and $\rho_{0}$ is the unit density (1000 kg m^−3^). A linear regression, forced through the origin, of ${\rho_{0}d}_{\text{ae}}^{2}C_{c}(d_{\text{ae}})$ versus $d_{m}^{2}C_{c}(d_{m})$ was performed, with the gradient of this linear regression corresponding to the effective mass density $(\rho_{e})\text{.}$ The description of the Cunningham slip correction factor used in this work was taken from Kim et al. (2005) and is given by:

(6)  Cc=1+lr(1.165+0.483exp{−0.997lr})

in which *ℓ* is the mean free path calculated by the SMPS and r is the mobility or aerodynamic particle radius (depending on whether $C_{c}(d_{m})$ or $C_{c}(d_{\text{ae}})$ is being calculated, respectively).

### 2.6. Particle collection and imaging by scanning electron microscopy

Samples of aerosol particles were collected on Cyclopore track-etched polycarbonate membranes (Whatman, Cat number 7060-4701) for scanning electron microscopy (SEM) imaging. The membranes were 47 mm in diameter with a pore diameter of 0.1 µm. Samples of interest were atomized and dried as described in Section 2.1. A mass flow controller connected to a vacuum pump was used to regulate the flow rate of the sample drawn from the generated dry aerosol plume to 0.9 L min^−1^. This flow rate was chosen to replicate the same aerosol sample residence times in the diffusion driers as for when we characterize aerosol optical and mass density properties. The aerosol samples were drawn through the polycarbonate filter contained within an in-line filter holder to facilitate collection for a 3-min sampling period. The membranes containing deposited aerosol samples were mounted on aluminum stubs and then sputter-coated with silver to make them conductive before they were imaged by a scanning electron microscope (Jeol, JSM-IT300) operating at 15.0 kV in high vacuum mode.

## 3. Results and discussion

In the following sections, we first evaluate the accuracy and sensitivity in measured extinction and absorption cross-sections (Section 3.1) and the precision in retrieved complex refractive indices (Section 3.2) for non-absorbing ammonium sulfate and absorbing nigrosin aerosol particles. We then use the retrieved complex refractive indices for aerosol particles comprised of internal mixtures of nigrosin with ammonium sulfate or sucrose to validate refractive index mixing rules (Section 3.3). Section 3.3 also discusses the accuracy in retrieved values for *n* and *k*. Finally, Section 3.4 examines the morphology of the aerosol particles from SEM images.

### 3.1. Experimental measurements of extinction and absorption cross-sections for pure components

Here, we use our spectroscopy measurements on aerosol particles comprised of ammonium sulfate or nigrosin to assess the accuracy and sensitivity in the measured extinction and absorption cross-sections for a non-light absorbing and strongly light absorbing species, by comparing our measured cross-sections with Lorenz-Mie predictions calculated using literature refractive indices. We also report measured extinction and absorption cross-sections for aerosol particles comprised of sucrose. However, the lack of reported literature refractive index values for sucrose in its dry and pure form precludes direct comparisons of our measurements with model predictions.

#### 3.1.1. Ammonium sulfate

**Figure 2.**
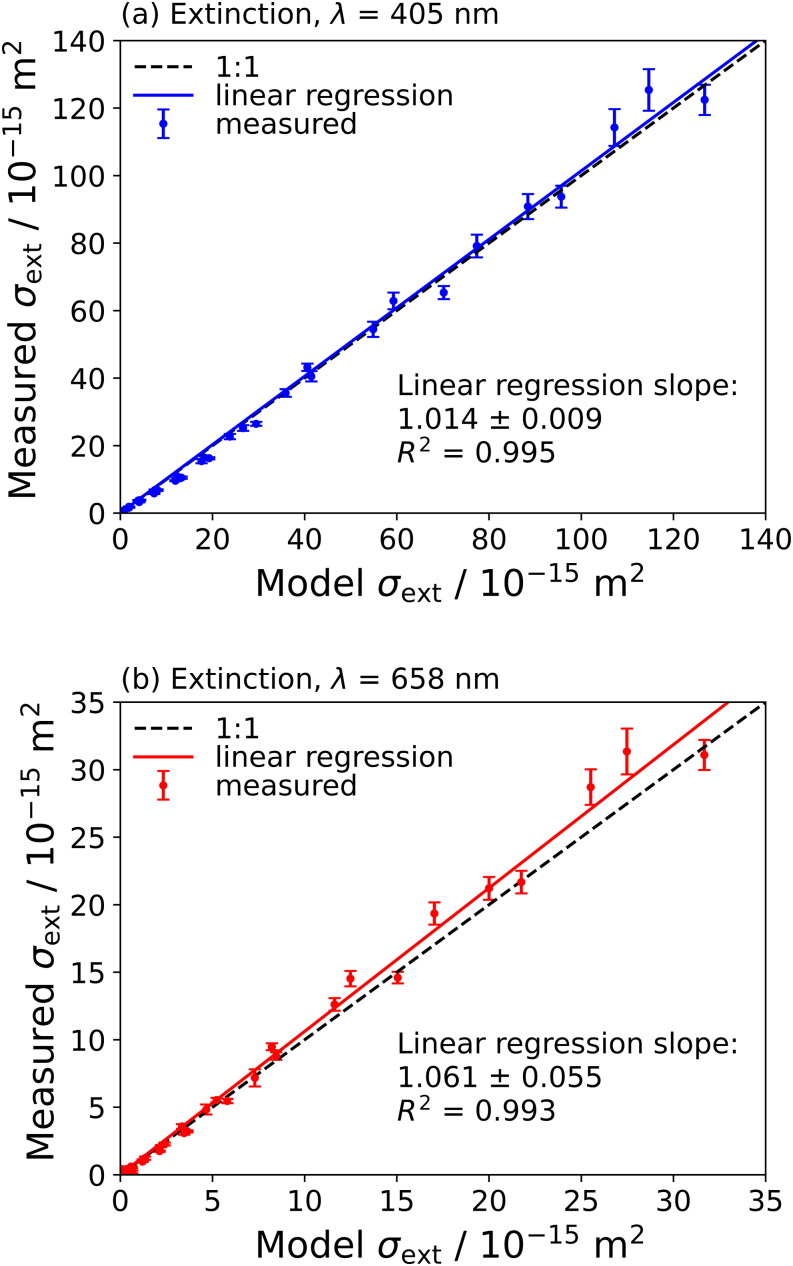
Comparison of measured and modeled extinction cross-sections for aerodynamically selected ammonium sulfate aerosol particles at optical wavelengths of (a) 405 nm, and (b) 658 nm. Model cross-sections are calculated using the real component of RI from a Cauchy curve fit to literature values (Cotterell et al. 2020). Dashed lines represent the 1:1 ratio between model and measured values and solid lines represent the linear regression of the measured versus modeled values forced through the origin. The slope of this regression and its standard error is also shown; the *R*^2^ value is the coefficient of determination.

Figures 2a and b show the measured extinction cross-sections at wavelengths of 405 nm and 658 nm, respectively, for three repeat measurements on ammonium sulfate aerosol particles, compared to corresponding values for the Lorenz-Mie modeled cross-sections. These three repeat measurements represent technical replicates of the measurement data, involving optical cross-section characterizations at the 13 different aerodynamic diameters, with each separate replicate dataset analyzed individually by the analysis discussed in Section 2.4. The model predictions used the *n* values for ammonium sulfate from a fit of the Cauchy equation to literature values performed by Cotterell et al. (2020). The best-fit parameters reported by the authors give *n* values at our measurement wavelengths of *n*_405_ = 1.5383 and *n*_658_ = 1.5174, which we used to calculate model cross-sections for comparison with our measured values. The linear regressions in Figure 2 were performed using a least-squares procedure to fit a straight line to the data forced through the origin, consistent with the linear regression analysis applied by Cotterell et al. (2020) for their analysis of mobility selected aerosol particle samples. The least squares-residuals were not weighted for measurement uncertainty and the slopes were 1.014 ± 0.009 and 1.061 ± 0.012 for the 405-nm and 658-nm extinction data, respectively. These near-unity values (particularly for the 405-nm measurements when the one standard deviation uncertainties in the slope are considered) indicate minimal levels of bias in our measured extinction cross-sections for ammonium sulfate aerosol particles. A version of Figure 2 with rescaled axes is given in Figure S2 of Section S6 of the SI, making it easier to resolve cross-section measurements at smaller sizes. Residual plots for the linear regressions plotted in Figure 2 are given in Section S7 of the SI. For completeness, we repeated the linear regressions allowing the intercepts to vary (i.e., with the intercept not forced through the origin). The p-values for these unforced intercepts were >0.05 and therefore not statistically significant. These intercepts, and their corresponding p-values are given in Section S10 of the SI.

Our ammonium sulfate extinction cross-section measurements, using an AAC for aerodynamic size selection, show greater precision than those reported by Cotterell et al. (2020) that used a DMA to select aerosol particles by their mobility diameter. A comparison of the measured and modeled cross-sections over the cross-section range of our measurements is shown in Figure S6 of the SI. The lower standard deviations for the measurements using AAC selection, compared to those using mobility selection, are a result of the higher transmission efficiency of the AAC providing higher particle number concentrations for downstream sampling by the CRDS spectrometers and CPC.

Figure S12 shows the measured absorption coefficient as a function of aerodynamic diameter for the three repeat measurements on pure ammonium sulfate aerosol particles at wavelengths of 405 nm and 658 nm respectively. Ammonium sulfate does not absorb light at visible wavelengths. Indeed, our experimental values of absorption coefficients are zero within one standard deviation. The average standard deviations across all three repeat measurements were 0.22 Mm^−1^ and 1.19 Mm^−1^ for the 405-nm and 658-nm measurements, respectively. The better precision of the 405-nm spectrometer may be caused by small variations in the performance of the microphones between the two spectrometers, or differences in the electronic noise in the separate circuit boards that are used to process the microphone signals.

#### 3.1.2. Nigrosin

**Figure 3.**
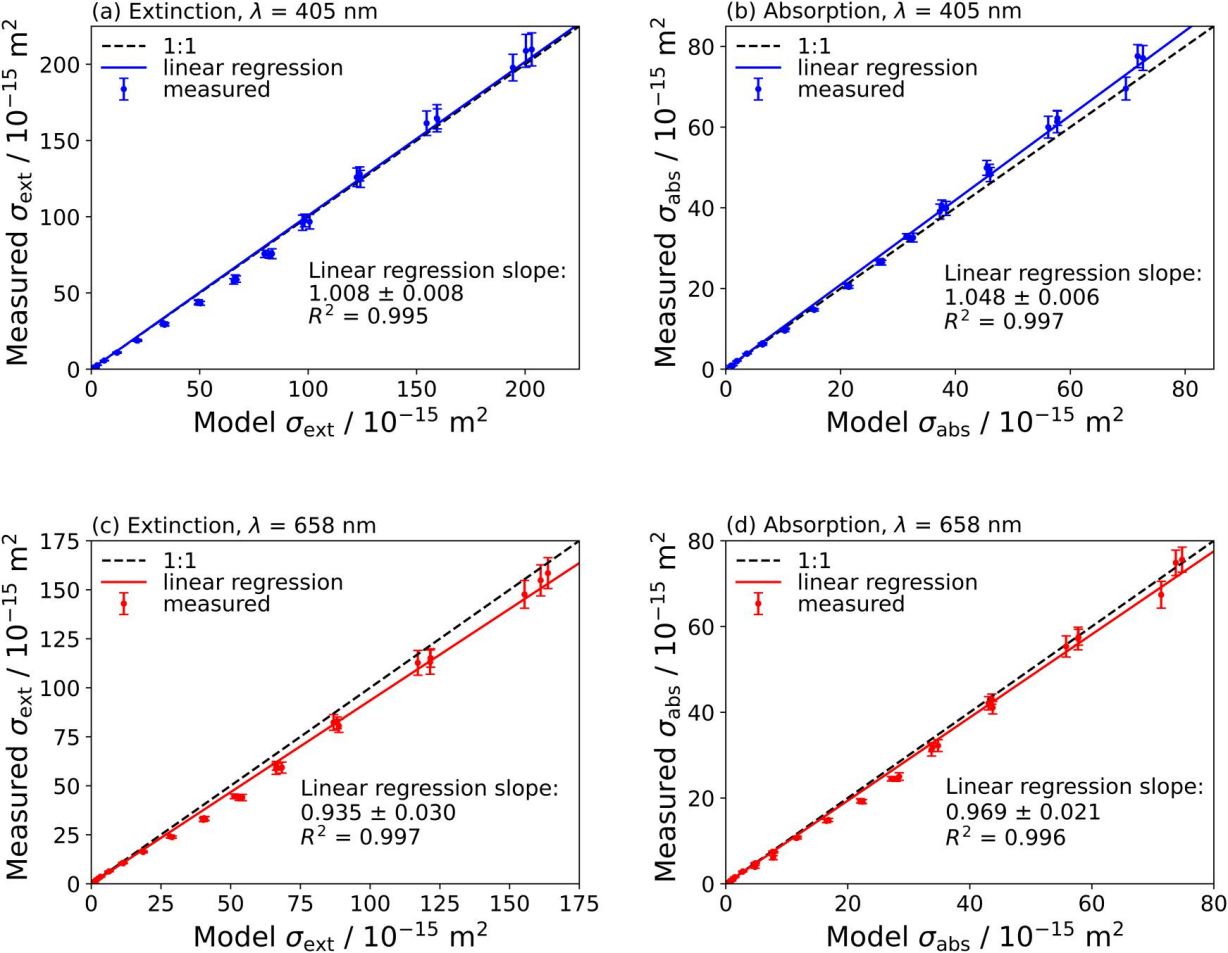
Comparison of measured and modeled cross-sections for aerodynamically selected nigrosin aerosol particles for extinction cross-sections measured at optical wavelengths of (a) 405 nm and (c) 658 nm, and absorption cross-sections measured at optical wavelengths of (b) 405 nm and (d) 658 nm. Model cross-sections are calculated using the complex refractive indices reported by Bluvshtein et al. (2017). Dashed lines represent a 1:1 ratio between model and measured values, and solid lines represent the linear regression of the measured versus modeled values forced through the origin. The *R*^2^ value is the coefficient of determination.

The analysis above for our measurements on non-absorbing ammonium sulfate aerosol particles are unable to quantify the accuracy of absorption cross-section measurements. Here, we report measurements for nigrosin aerosol particles, with nigrosin used commonly as a benchmark species for characterizing the performance of instruments that measure aerosol light absorption, although the composition of nigrosin dye *might* vary between manufacturers and batches (Sedlacek and Lee 2007). Since nigrosin dye is a mixture of components the effective mass density is not necessarily constant between batches, indeed a range of values have been reported including 1600 kg m^−3^ (Moteki et al. 2010) and 1340 kg m^−3^ (Radney and Zangmeister 2015). The extinction and absorption cross-sections for three repeat measurements on nigrosin aerosol particles are shown in Figure 3. Model cross-sections are calculated using the complex RI values reported by Bluvshtein et al. (2017) of *n*_405_ = 1.624 ± 0.0063, *k*_405_ = 0.1541 ± 0.0081, *n*_658_ = 1.811 ± 0.0067 and *k*_658_ = 0.2476 ± 0.0031. Linear regressions of the measured versus model cross-sections, shown on the figures, are forced through the origin. There is closer agreement for the 405-nm compared to the 658-nm extinction cross-sections, with the regression slopes for σ_ext_ taking values of 1.008 ± 0.008 and 0.935 ± 0.030 for the 405-nm and 658-nm measurements, respectively. The measured extinction cross-sections for ammonium sulfate show closer agreement to the model values than those for nigrosin; the literature values of the refractive indices for ammonium sulfate are quantified accurately and it has a well-defined composition, in contrast to the ill-defined composition of nigrosin that might differ between the batch used here and that used by Bluvshtein et al. (2017). It has been shown that the absorption spectrum of nigrosin exhibits a weak pH spectral dependence (Radney and Zangmeister 2015) which could bias the comparison to the values from Bluvshtein et al. (2017).

Figures 3b and d compare our measured absorption cross-sections for nigrosin particles with those predicted by our Lorenz-Mie model. The slopes of these distributions, from linear regressions forced through the origin, are 1.048 ± 0.006 for the 405-nm and 0.969 ± 0.021 for the 658-nm measurements. Again, the 4.8% and 3.1% systematic errors indicated by these slopes may be indicative of differences in composition between our nigrosin batch with that interrogated by Bluvshtein et al. (2017). The discrepancies in measured absorption cross-sections may also arise from errors introduced during the PAS calibration process. However, our PAS instruments were calibrated with ozone-laden air samples, the validity of which was demonstrated in several publications (Davies et al. 2018; Cotterell et al. 2021, 2019a) and this is unlikely to be the reason for differences. Particle generation methods are also expected to affect optical properties; the *n* and *k* values reported by Bluvshtein et al. (2017) were derived from spectroscopic measurements on thin films of solid nigrosin, in contrast to the measurements used in this work that involved atomization from aqueous solutions and subsequent rapid particle drying. Expanded scale plots for these linear regressions are given in Section S6 and residual plots are given in Section S7 of the SI. For completeness, we repeated the linear regressions for the extinction and absorption cross-section datasets allowing the intercepts to vary, and the p-values were statistically significant. The coefficients for these linear regressions, along with the p-values for the intercepts, are provided in Section S10 of the SI. The precision of absorption and extinction cross-sections measured for nigrosin is improved when using an AAC compared to a DMA for size selection. Figure S7 in the SI compares the measured optical cross-sections with those from Cotterell et al. (2020), with this improvement in precision associated with the higher transmission efficiency of the AAC.

#### 3.1.3. Sucrose

The extinction cross-sections for sucrose aerosol particles as a function of the mean measured mobility diameter for a representative data set are shown in Figure S8 of the SI. We are not aware of any reported refractive indices for pure sucrose in its dry form, and so modeled cross-sections have not been calculated for comparison. The corresponding measured absorption coefficients for a representative data set are shown in Figure S9 of the SI, demonstrating an absorption coefficient of ∼0 Mm^−1^ within one standard deviation as expected. The mean standard deviation in absorption coefficients are 0.18 Mm^−1^ and 1.06 Mm^−1^ for the 405-nm and 658-nm measurements, respectively, which are comparable to those reported above for measurements on ammonium sulfate aerosol particles.

### 3.2. Complex refractive index retrievals for pure components

The measured optical cross-sections from Section 3.1 are used to retrieve the complex RI of the pure component aerosol particles using the methodology described in Section 2.4. Complex refractive indices were retrieved by fitting the extinction and absorption cross-section measurements to Lorenz-Mie calculations simultaneously; this approach differs from other studies that have used an extinction-only retrieval. Table 1 summarizes the mean values of the retrieved complex refractive indices (from three repeat retrievals) for aerosol particles composed of ammonium sulfate, nigrosin and sucrose.

**Table 1.** Summary of refractive index retrievals for pure component aerosol particles comprised of ammonium sulfate, nigrosin and sucrose measured at optical wavelengths of 405 nm and 658 nm. The uncertainties represent one standard deviation obtained from three repeat retrievals.

Species	*n*_405_	*k*_405_	*n*_658_	*k*_658_
ammonium sulfate	1.518 ± 0.018	0.0001 ± 0.0001	1.517 ± 0.017	0 ± 0
nigrosin	1.583 ± 0.015	0.1539 ± 0.0033	1.713 ± 0.030	0.2345 ± 0.0065
sucrose	1.513 ± 0.010	0.0001 ± 0.0071	1.508 ± 0.001	−0.0007 ± 0.0036

**Figure 4.**
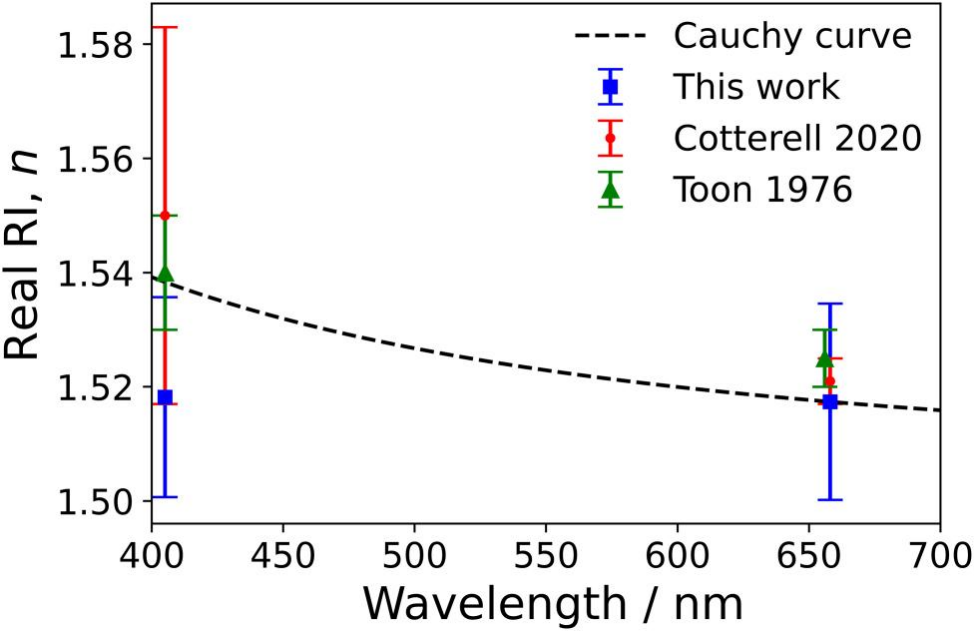
Retrieved real refractive indices for aerodynamically selected ammonium sulfate aerosol at 405 nm and 658 nm wavelengths (blue squares) compared to values utilizing mobility selection reported by Cotterell et al. (2020) (red circles), and transmission measurements on macroscopic (∼centimeter scale) ammonium sulfate crystals by Toon, Pollack, and Khare (1976) (green triangles). Also shown is the Cauchy curve (black dashed line) obtained by Cotterell et al. (2020) through a fit to *n* values from eight different literature studies. Error bars represent one standard deviation in the reported *n* values.

The refractive indices for ammonium sulfate retrieved here are compared in Figure 4 to values retrieved from mobility-selected data as reported by Cotterell et al. (2020), as well as the best fit of the Cauchy curve to eight other literature studies performed by the same author. Real RI values reported by Toon, Pollack, and Khare (1976) are also shown for comparison since these were measured at similar wavelengths. Our reported mean *n*_405_ value is 1.3% lower than that obtained from the Cauchy fit and our *n*_658_ is the same as the Cauchy value. Meanwhile, our *n*_405_ and *n*_658_ values are 1.4% and 0.5% lower, respectively, than the values reported by Toon, Pollack, and Khare (1976). While the refractive indices reported by Toon, Pollack, and Khare (1976) are often used as a benchmark for ammonium sulfate, it should be noted that these correspond to macroscale crystals grown over the course of a year rather than nanoscale particles atomized from solution and rapidly dried. The real components reported by Cotterell et al. (2020) for mobility selected ammonium sulfate aerosol are *n*_405_ = 1.550 ± 0.033; and *n*_658_ = 1.521 ± 0.004, with our *n*_405_ and *n*_658_ values lower than these mobility-based retrievals by 2.1% and 0.26%, respectively. Our *k*_405_ of 0.0001 is larger than 10^−7^ and non-zero but represents the smallest step size used in the grid search algorithm. These levels of agreement with different literature values are expected for ammonium sulfate, which has a well-defined composition. Resolving the weak dispersion of *n* for ammonium sulfate, shown by the Cauchy curve, at these wavelengths is outside the sensitivity of our measurement approach.

It is important to note that the biases in *n* indicated in Figure 4 do not connect directly with those in the simple linear regression slopes shown in Figure 2. For example, the *n* value at 405 nm shown in Figure 4 is 1.3% lower than the best-fit Cauchy curve, while the simple linear regression in Figure 2 indicates that the measured cross-sections are 1.4% larger than the predicted cross-sections using the literature value for refractive index. The regressions in Figure 2 represent a simple least-squares linear regression to the plotted cross-sections in each panel, fitting a straight line (forced through the origin) to the extinction and absorption cross-section data separately, and are consistent with the analysis presented in our previous publication that examined measurements for mobility classified aerosol particles (Cotterell et al. 2020). Meanwhile, the RI retrievals use a merit function that includes a weighting for the variance in the measured cross-sections, as described in Section 2.4, to fit simultaneously the measured extinction and absorption cross sections to Lorenz-Mie theory. Arguably, a better least-squares regression approach for treating the data in Figure 2 is to use least-squares residuals that are weighted for the variance in the measured cross-sections, in a way that is more consistent with the merit functions used for RI retrievals. These alternative regression plots are provided in Section S10 of the SI and indicate slopes that are more consistent with the directions and levels of bias seen in the retrieved *n* in Figure 4.

The average percentage standard deviations across three repeat retrievals in *n*_405_ and *n*_658_ for ammonium sulfate are 1.15% and 1.13%, respectively. In contrast, the percentage standard deviations in *n*_405_ and *n*_658_ reported by Cotterell et al. (2020) from five repeat measurements on mobility-selected particles are 2.19% and 0.26%, respectively. Our experiments had a CPC sampling directly from the exhaust of the 405-nm PAS cell, while the mobility measurements were performed with a CPC sampling from the exhaust of the 658-nm PAS cell. These uncertainty values indicate that more precise RI retrievals are obtained when attenuation coefficient and number concentration measurements are measured concurrently in a serial flow configuration, and that further uncertainty is introduced when relying on a transmission factor for determining particle number concentrations.

**Figure 5.**
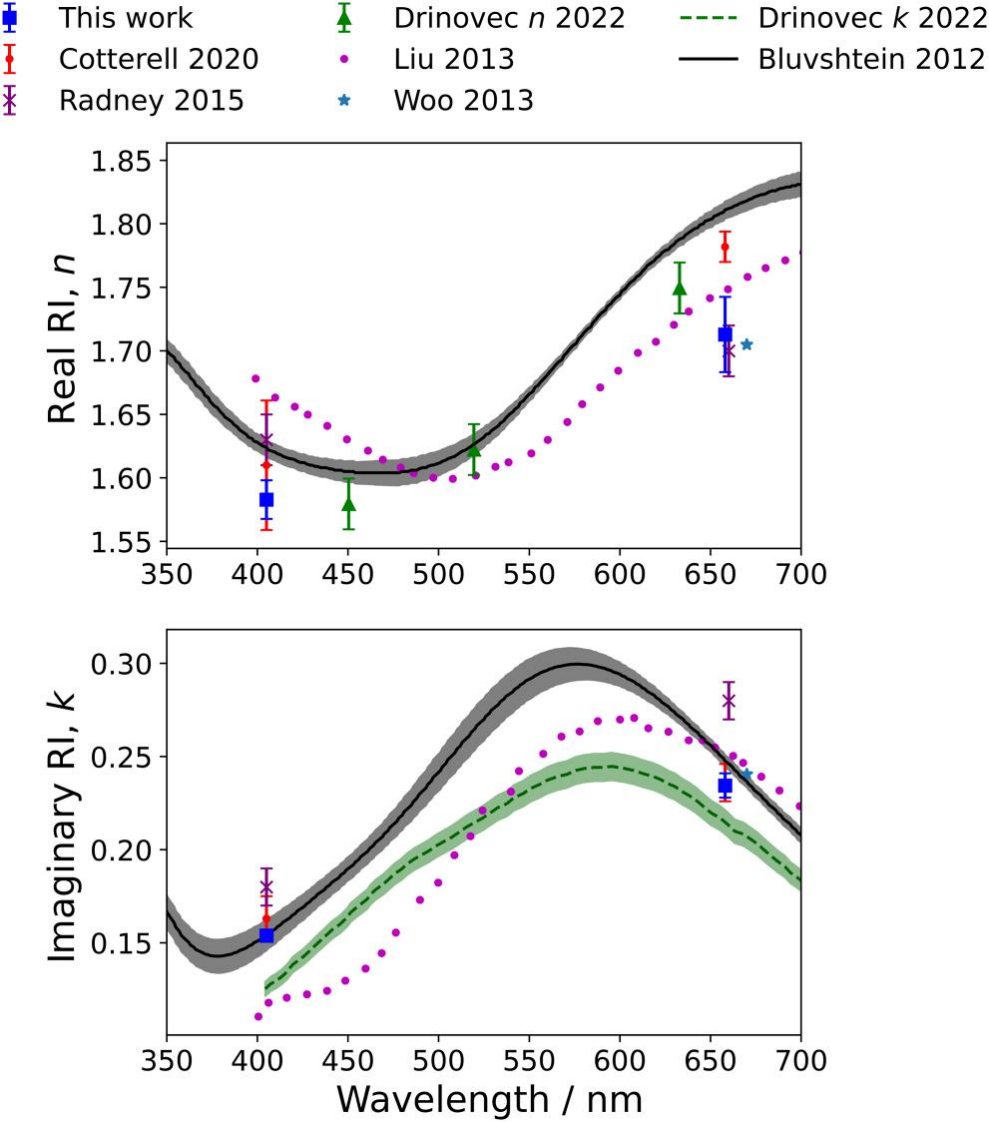
Retrieved real and imaginary refractive indices for aerodynamically selected nigrosin aerosol particles at 405 nm and 658 nm wavelengths (blue squares) compared to mobility selected nigrosin aerosol particles from Cotterell et al. (2020) (red circles), Radney and Zangmeister (2015) (purple crosses) and Woo, You, and Lee (2013) (light blue stars). Also shown are measurements using thin films of nigrosin (Liu, Zhang, and Martin 2013; Bluvshtein et al. 2017; Drinovec et al. 2022). Error bars represent one standard deviation in the reported *n* and *k* values where reported. The envelopes (grey and green) represent one standard deviation in the measurements reported by authors.

The mean refractive indices retrieved for nigrosin aerosol particles are given in Table 1. Figure 5 compares our retrieved *n* and *k* values for nigrosin to mobility selected nigrosin aerosol particle measurements from three different aerosol studies at similar optical wavelengths to our measurements (Woo, You, and Lee 2013; Radney and Zangmeister 2015; Cotterell et al. 2020). Also shown for comparison are measurements from three studies characterizing the optical properties of thin films of nigrosin (Liu, Zhang, and Martin 2013; Bluvshtein et al. 2017; Drinovec et al. 2022). Our retrieved values lie within the range of reported literature values. Differences between measured values at similar wavelengths might be caused by variations in the compositions, and therefore the optical properties, of nigrosin that might arise between different batches. The reported batch numbers for our aerosol measurements, those by Cotterell et al. (2020) and the spectroscopic ellipsometry measurements from Bluvshtein et al. (2017) are different. In addition, the methods for preparing nigrosin samples could affect the optical properties that are measured. The optical properties of thin films of nigrosin may contrast to the aerosol measurements we report. The methods for preparing aerosolized nigrosin samples are also different between this work and in the mobility selected aerosol studies (Woo, You, and Lee 2013; Radney and Zangmeister 2015; Cotterell et al. 2020), which used a cross-flow atomizer.

As discussed above, the smaller one standard deviation uncertainty and greater precision in RI for the 405-nm retrievals is likely attributed to the CPC measuring number concentration directly from the 405-nm sampling line instead of relying on a transmission factor correction, the value of which may be affected by small variations in operating conditions. Cotterell et al. (2020) observed smaller average percentage uncertainties in determined RI values from 658-nm measurements (that featured a CPC sampling from the exhaust of the 658-nm PAS cell) compared to those at 405 nm (that relied on a transmission factor correction). The authors observed average percentage uncertainties for *n*_405_, *k*_405_, *n*_658_ and *k*_658_ of 3.2%, 7.4%, 0.67% and 4.2%. In contrast, we observe corresponding average percentage uncertainties of 0.95%, 2.1%, 1.8%, and 2.8% for nigrosin aerosol particles and see smaller percentage uncertainties in the 405-nm values. When considering the change in CPC location between the two studies, our approach using an AAC allows for better precision in retrieved refractive indices. The accuracy in *n* and *k* was reported to depend strongly on biases in the number concentration measured by the CPC (Cotterell et al. 2020). Monte Carlo simulations showed a 5% bias in number concentration resulted in a decrease in accuracy for complex RI retrievals of up to 5.3%, with *k* demonstrating a larger degradation in accuracy than *n*. The authors also reported that a 5% bias in the measured PAS calibration coefficients introduces an error of ∼9% in the retrieved *k* values for nigrosin at both wavelengths. Other sources of systematic error include the uncertainty in the ratio of the length of the CRDS cavity to the length occupied by the aerosol sample (*R*_L_), although the authors found this to have minimal impact on the retrieval accuracy. The systematic errors in mobility diameter obtained from the SMPS were assessed by Kinney et al. (1991), who determined the uncertainty to be ±3%. The mobility size is used during calculations of model cross-section values to compare to the measured values during the grid search retrieval process and so this uncertainty will also introduce systematic error in the retrieved RI values.

The retrieved refractive indices for aerosol particles comprised of pure sucrose are also given in Table 1. To our knowledge, this is the first time that refractive indices for dry sucrose aerosol particles have been reported and so no comparison to literature values is made. The low values for the standard deviation (expressed as a percentage of the mean) of 0.69% and 0.053% for retrievals of *n*_405_ and *n*_658_ respectively demonstrate high levels of precision in our retrievals. The negative values of *k* indicated in Table 1 for sucrose represents a limitation of our grid search algorithm, as we do not constrain k to be positive, resulting in these unphysical negative values.

### 3.3. Complex refractive indices for mixtures

This section reports the retrieved refractive indices for binary, internally mixed aerosol particles comprised of non-absorbing ammonium sulfate or sucrose mixed with light-absorbing nigrosin dye. The mixing behavior of organic nigrosin with an inorganic species (ammonium sulfate) is contrasted to mixing nigrosin with another organic species (sucrose), and the variations in the particle complex refractive indices and effective densities with changing mass ratios is compared for the two mixture types.

The retrieved *n* and *k* values across the mixing range are used to challenge refractive index mixing rules, including the linear mass fraction weighting of refractive index (or *mass fraction mixing*) and mole fraction weighting of molar refraction (or *molar refraction mixing*) models (Liu and Daum 2008). The mass fraction mixing rule takes a mass fraction weighting of the complex refractive indices of the pure components to predict the RI of the mixture and assumes ideal mixing between the two components. The mass fraction mixing rule to calculate the real and imaginary refractive indexes is given by Equations (7) and (8), respectively, for a binary mixture comprised of two species A and B with known mass fractions *w*_A_ and *w*_B_ and having pure component refractive indices ($n_{A},k_{A}$) and ($n_{B},k_{B}$).

(7) $$n_{\text{mix}}=w_{A}n_{A}+(1-w_{A})n_{B}$$

(8) $${\;k}_{\text{mix}}=w_{A}k_{A}+(1-w_{A})k_{B}\;$$

In contrast, the molar refraction mixing rule has an underlying physical basis and accounts for the densities of the pure components and the mixed particles. The molar refraction of a species (*R*) is defined by:

(9) $$R=(\frac{m^{2}-1}{m^{2}+2})\frac{M}{\rho }$$

in which *M* is the molecular weight, and ρ is the effective mass density. The pure component particle effective mass densities were measured using the approach described in Section 2.5 and were (1896 ± 18, 1563 ± 10, and 1527 ± 16) kg m^−3^ for ammonium sulfate, sucrose, and nigrosin, respectively. The effective density of ammonium sulfate was higher than the bulk density of 1773 kg m^−3^ (Johnston and Adams 1912). This bias is consistent with assessments made by Vokes et al. (2022) where the overall accuracy of our approach to density retrievals was estimated to be 7.1%. The pure component molecular weights used were 132 g mol^−1^ for ammonium sulfate and 342 g mol^−1^ for sucrose. The molecular structure for nigrosin is not defined precisely and a range of molecular formulas and molecular weights for nigrosin have been reported, with the latter ranging from 616.5 g mol^−1^ to 1090 g mol^−1^ (Lack et al. 2006; Hasenkopf et al. 2010; Khalaf et al. 2022). We have chosen an intermediate value of 820 g mol^−1^ for calculations. The actual values of molecular weights used for the pure components are not important since the molecular weight terms cancel when calculating refractive indices for the mixtures. The molar refraction of a binary mixture comprised of two species A and B with known mole fractions *x*_A_ and *x*_B_ is calculated from their corresponding molar refractions (*R*_A_, *R*_B_) using:

(10) $$R_{\text{mix }}=x_{A}R_{A}+{(1-x}_{A})R_{B}$$

Finally, the complex refractive index of the mixture (*m*_mix_) is obtained from *R*_mix_ using Equation (12) and the effective mass density ($\rho_{\text{mix}})$ and molecular weight (*M*_mix_) of the mixture. $\rho_{\text{mix}}$ is determined from parameterization of experimentally measured values (see below), while *M*_mix_ is calculated through conservation of mass by weighting the pure component molecular weights by their corresponding mole fractions as defined in Equation (11).

(11) $$M_{\text{mix}}=x_{A}M_{A}+(1-x_{A})M_{B}$$

(12) $$m_{\text{mix }}=\sqrt{\frac{M_{\text{mix}}+2R_{\text{mix}}\rho_{\text{mix}}}{M_{\text{mix}}-R_{\text{mix}}\rho_{\text{mix}}}}$$

In Section S13 of the SI, we explore the molar refraction mixing rule more closely. Importantly, we show that when this predictive framework of the molar refraction mixing rule is applied to light absorbing particles, *n*_mix_ depends on *both* the real and imaginary components of the pure components, and similarly for *k*_mix_. The prediction of *n*_mix_ and *k*_mix_ must not be treated separately for light absorbing substances, otherwise considerable errors may occur.

The effective mass density of a binary mixture of A and B can be predicted using an ideal mixing rule that assumes mass and volume are conserved on mixing (Vokes et al. 2022):

(13) $$\rho_{\text{mix},\text{ideal}}=\frac{1}{\frac{w_{A}}{\rho_{A}}+\frac{\left({1-w}_{A}\right)}{\rho_{B}}}$$

in which *w*_A_ is the mass fraction of component A, and *ρ*_A_ and *ρ*_B_ are the pure component densities. This ideal mixing rule is suitable for materials that behave ideally and show no change in total volume on mixing. However, species often mix in a non-ideal way causing a change in the volume of the mixture and resulting in a deviation in mass density away from that predicted by Equation (13). Instead, we used measurements of the mobility diameter of the aerosol particles in combination with the aerodynamic diameter to calculate effective mass densities, using the methodology described in Section 2.5.

**Figure 6.**
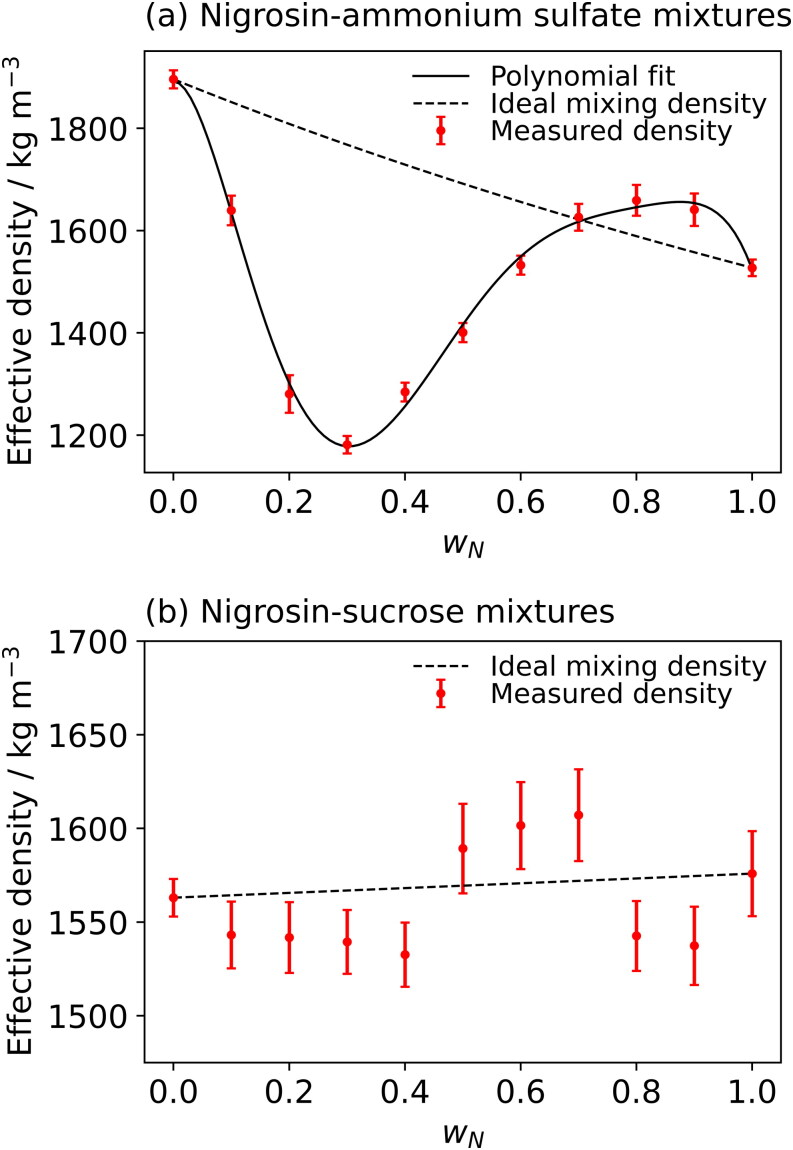
Effective densities determined from the aerodynamic and mobility diameters of: (a) nigrosin-ammonium sulfate mixtures; and (b) nigrosin-sucrose mixtures. Dashed lines show the predicted densities assuming ideal mixing, constrained by the measured pure component values. The solid line in (a) shows a polynomial fit to the experimental data as described in the main text. Error bars represent the standard error in the linear regression performed when calculating the mass density.

Figure 6 shows the experimentally measured effective densities for nigrosin-ammonium sulfate and nigrosin-sucrose mixtures. The effective mass density of pure nigrosin measured here was (1527 ± 16) kg m^−3^. This compares with values of (1632 ± 16) kg m^−3^ reported by Vokes et al. (2022) and (1500 ± 60) kg m^−3^ reported by Radney and Zangmeister (2018). The predicted ideal mass density using Equation (13) is shown for both systems. The effective mass densities for mixtures of nigrosin and ammonium sulfate show a non-linear relationship with nigrosin mass fraction, and the ideal mixing treatment fails to predict accurately the mixture densities. Instead, a 6^th^ order polynomial is fit to the effective densities to parameterize them for inclusion in the molar refraction mixing rule:

(14) $$\rho_{\text{mix}}=a{w_{N}}^{6}+b{w_{N}}^{5}+c{w_{N}}^{4}+d{w_{N}}^{3}+e{w_{N}}^{2}+f$$

where *w*_N_ is the mass fraction of nigrosin and the parameters *a*, *b*, *c*, *d*, *e* and *f* took the values of (−88500 ± 11740, 287500 ± 32780, −353500 ± 33320, 196100 ± 14670, −42000 ± 2416 and 1892 ± 18) kg m^−3^ as determined by a least-squares fit of Equation (14) to the measured mass density values. Mixing of ammonium sulfate and nigrosin results in non-ideal mixing, with the volume change of mixing lowering the density below that predicted by the ideal mixing model at low values of *w*_N_. In contrast, mass densities of mixtures of the organic species, nigrosin and sucrose, showed a near-linear dependence on nigrosin mass fraction and the ideal mixing rule predicts the general trend in effective mass densities. Figures S13 and S14 in the SI summarize the distributions in measured effective mass densities with mixture composition for a broad range of two-component mixtures, including organic-inorganic and organic-organic mixtures. Our studies so far indicate that it is difficult to draw trends with regards to whether mixing will cause significant deviations from ideal mixing, although we observe considerable deviations from ideal mixing for nigrosin mixtures with all the inorganic species explored.

Extinction and absorption cross-section measurements, for aerosol particles comprised of mixtures of nigrosin with either ammonium sulfate or sucrose, were used to retrieve the complex refractive index; we performed measurements on two-component mixtures with nigrosin mass fractions ranging from 0 to 1 in 0.1 increments. Three repeat retrievals were carried out for pure components and average values for the standard deviations, expressed as a percentage of the mean, for 405-nm and 658-nm measurements were calculated to allow estimation of the standard deviations in the single repeat measurements on two-component mixtures.

**Figure 7.**
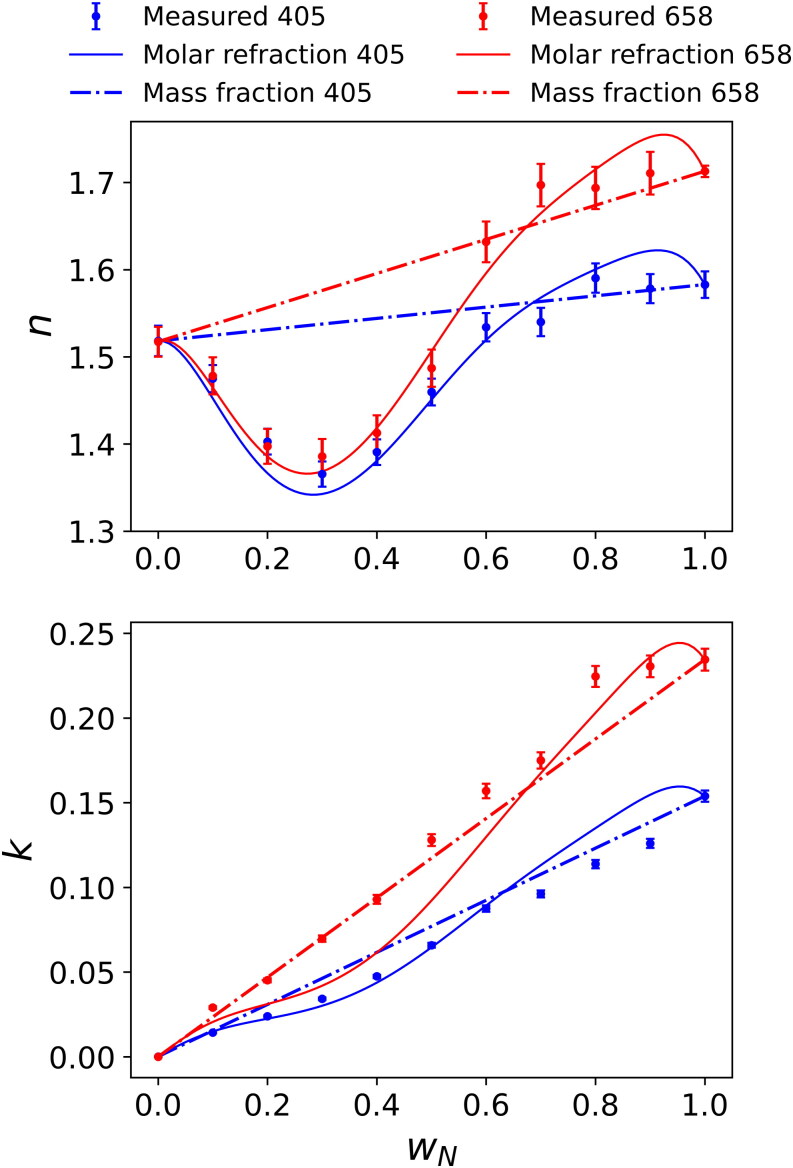
The retrieved *n* and *k* values (at wavelengths of 405 and 658 nm), for mixtures of ammonium sulfate and nigrosin, versus the mass fraction of nigrosin (*w*_N_). Error bars represent one standard deviation in the retrieved values. Solid lines represent the predictions of *n* and *k* using the molar refraction mixing rule, incorporating the measured effective mass density. Dashed lines represent predictions of *n* and *k* using the mass fraction mixing rule.

Figure 7 shows the experimentally retrieved *n* and *k* values for the two-component mixtures of nigrosin with ammonium sulfate. The figure also shows predictions from both the molar refraction and mass fraction mixing rules at the two wavelengths used. The retrieved *n* values show highly non-linear behavior with nigrosin mass fraction, and the mass fraction mixing rule prediction fails to capture this non-linear trend. Instead, the molar refraction mixing rule should be used to account for the effects of non-ideal mixing on variations in mass density when mixing nigrosin and ammonium sulfate. The molar refraction mixing model uses the density parameterization for the experimental measurements shown in Figure 6a. Retrieved values for *k* show a near-linear relationship with nigrosin mass fraction and the experimental data is captured well by the mass fraction mixing rule for the data recorded at both wavelengths. The molar refraction mixing rule captures the broad trend in the retrieved values for *k* with mixture composition. However, there are notable discrepancies between our retrieved *k* and predicted values from the molar refraction mixing rule at some mixture compositions. We hypothesize that one source of this discrepancy is the impact of the uncertainty in measured effective mass density, and the accuracy of parameterization of this data, on the molar refraction mixing rule predictions. For example, Vokes et al. (2022) report an overall accuracy in effective mass density of 7.1% using the same measurement approach. Moreover, our RI retrievals utilizing Lorenz-Mie theory and our method for determining particle densities assumed that the interrogated aerosol particles were spherical and chemically homogeneous. Electron microscopy imaging (see Section 3.4) provides limited evidence that our particles become less spherical at intermediate organic-inorganic mixing ratios that might contribute to the discrepancies observed in Figure 7. Meanwhile, the analysis methods available to us could not inform us of the degree of intra-particle chemical heterogeneity.

**Figure 8.**
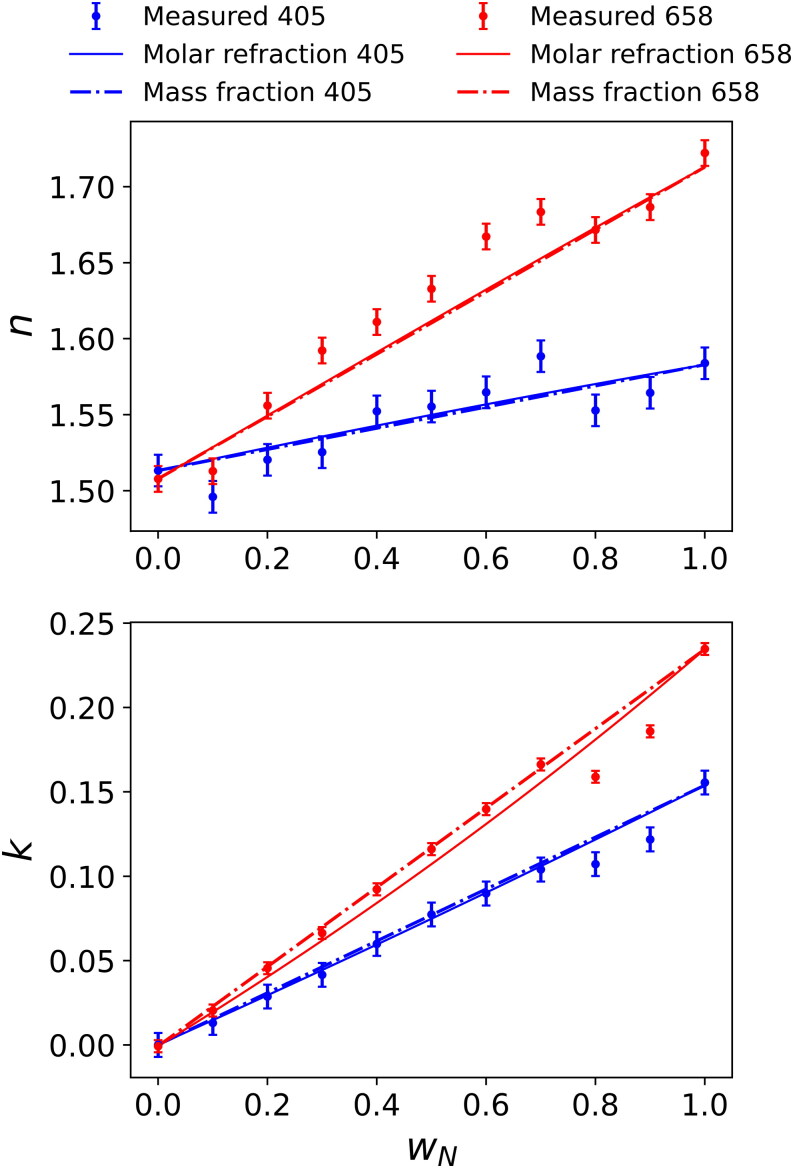
The retrieved *n* and *k* values (at wavelengths of 405 and 658 nm) for mixtures of sucrose and nigrosin, versus the mass fraction of nigrosin (*w*_N_). The uncertainty bars represent one standard deviation in retrieved values. Solid lines represent the predictions of *n* and *k* using the molar refraction mixing rule incorporating the ideal mixing description of particle densities. Dashed lines represent the predictions of *n* and *k* using the mass fraction mixing rule.

Figure 8 compares retrieved refractive indices for sucrose-nigrosin mixtures with molar refraction and mass fraction mixing rule predictions. For sucrose-nigrosin mixtures, the ideal mixing rule (as shown in Figure 6b) represents well the measured variations in effective densities with mixing ratio and is used in the molar refraction mixing calculations shown in Figure 8. The small levels of discrepancy between the retrieved and predicted values for *n* (of up to 0.03) are within the uncertainty associated with the accuracy of the retrieved values. For example, the analysis summarized in Table 1 of Cotterell et al. (2020) indicates that *a* ∼2% error in the retrieved *n* (corresponding to an absolute error in *n* of ∼0.03 for the values in Figure 8) arises from the typical uncertainties of ∼5% in number concentration measurements using a CPC. Additional factors (such as the effective length of the optical cavity of the CRDS spectrometer occupied by the aerosol sample) make further contributions to this uncertainty. A similar analysis for *k* is more complicated, with Table 1 of Cotterell et al. (2020) showing that the accuracy of *k* scales with the magnitude of *k* itself, from a 1% error in the limit of low absorption for ammonium sulfate to an error of ∼5% in the limit of strong absorption by pure nigrosin aerosol particles. In contrast to the mixtures with ammonium sulfate, the sucrose-nigrosin mixtures exhibit near-linear relationships for *n* and *k* with nigrosin mass fraction and both the mass fraction and molar refraction mixing rules produce near-identical trends in the complex refractive indices.

### 3.4. Morphology of aerosol particles

The morphology of aerosol particles generated and dried as described in Section 2.1, before size selection with the AAC, was investigated using SEM. Particles were collected and imaged using the method described in Section 2.6. Images of pure nigrosin and ammonium sulfate aerosol particles and selected mixtures of the two, collected on polycarbonate membranes, are shown in Figure S16. The pores of the polycarbonate membrane, with a diameter of 100 nm, are seen as dark circles and aerosol particles as light-colored areas. These images generally show particles with a spherical shape for pure nigrosin and ammonium sulfate. However, we acknowledge that these two-dimensional images of a limited number of particles do not provide a solid basis for quantification of particle shape, or changes in shape with internal mixing, and future work should couple microscopy with image analysis methods for statistical quantification of shape factors. Our determinations of particle effective mass density and retrievals of RI using Lorenz-Mie theory assume the particles adopt a spherical morphology. The apparent loss of sphericity for the mixed particle compositions may account for deviations of the predicted *k* from the molar refraction mixing rules seen for some mixtures. It is important to note that inhomogeneity in the compositions for the mixed particles, containing ammonium sulfate and nigrosin, could contribute to the observed discrepancies between measured and predicted values of RI using the molar refraction mixing rule. However, the electron microscopy imaging methods available to us were not able to measure spatial distributions in chemical composition. Exploring potential methods for examining intra-particle chemical composition distributions will be an important avenue for future work.

## 4. Conclusions

We have shown that the precision in measured optical cross-sections is improved considerably when using an AAC, in comparison to measurements using a DMA, to size-select particles on their electrical mobility, owing to the higher transmission efficiency of the AAC. Further improvements in the precision and accuracy of retrieved refractive indices, from fitting Lorenz-Mie theory to measured extinction and absorption cross-sections, might be realizable from alternative choices to the $\chi^{2}$ minimization approach.

This work has demonstrated some of the complexities that exist for two-component aerosol systems and shown the importance of determining particle effective densities for refractive index predictions. Further work from these observations could include studies to understand the generality of these findings, in binary mixtures and more complex multi-component systems. Future work should also examine further the mixing scenarios for when non-ideal interactions are expected to have significant impacts on densities and optical properties, as the results of this study are too limited to provide general insight into when non-ideal interactions are expected to be significant. In particular, extending our assessments to mixed particles containing water could improve significantly our understanding of light interactions with atmospheric aerosol particles. Future work could also involve applying the methods here to black carbon mimics to improve understanding of models for the optical properties of internally mixed black carbon particles.

## Supplementary Material

Supplemental Material

## Data Availability

Data are available at the University of Bristol data repository at https://doi.org/10.5523/bris.p75j112xrzs31zlmj3aa3ca59. For enquiries related to the data included in this paper, please contact Michael I. Cotterell (michael.cotterell@chem.ox.ac.uk).
