# Supplementary material for: An innovative approach to characterizing the refractive indices and effective densities of internally mixed light-absorbing aerosol particles
Source: Aerosol Sci Technol. 2025 Mar 6;59(7):857–76. doi: 10.1080/02786826.2025.2468966 (PMC13054925; doi:10.1080/02786826.2025.2468966)
Supplement: Supplemental Material [file UAST_A_2468966_SM0877.pdf]

Supplementary Information for "An Innovative Approach to Characterizing the Refractive Indices and Effective Densities of Internally Mixed Light-Absorbing Aerosol Particles"

Gwen R. Lawson,[1] Simon Xi Chen,[1] Guy Collins,[1] Naomi Lawson,[1] Kate Szpek,[2] James Bowles,[2] James Allan,[3] Justin M. Langridge,[2] and Michael I. Cotterell[3]*

[1]School of Chemistry, University of Bristol, Bristol, UK, BS8 1TS

[2]Met Office, Exeter, UK, EX1 3PB

[3]Department of Earth and Environmental Sciences, University of Manchester, Manchester, UK, M13 9PY

[4]Department of Chemistry, University of Oxford, Oxford, UK, OX1 3QZ

*Correspondence to:

Michael I. Cotterell (michael.cotterell@chem.ox.ac.uk)

Contents

S1. Calibration of AAC and SMPS

S2. Cavity ring-down spectroscopy

S3. Photoacoustic spectroscopy

S4. CPC transmission factors

S5. Example SMPS data

S6. Expanded scales for measured and modelled cross-section plots

S7. Residual plots for ordinary least-squares linear regressions

S8. Comparison of precision in cross-section measurements using mobility and aerodynamic size selection

S9. Sucrose optical cross-sections

S10. Comparison of ordinary and weighted least-squares linear regressions

S11. Absorption coefficients of ammonium sulfate

S12. Effective mass density characterizations for a range of two-component mixtures

S13. Further examination of the molar refraction mixing rule

S14. Scanning electron microscopy images of particles

S1. Calibrations of AAC and SMPS

Calibration of the SMPS and AAC was achieved using National Institute of Standards and Technology (NIST)-traceable polystyrene spheres (PSS, Thermo Scientific, 3000 Series Nanosphere Size Standards) with nominal diameters of (80, 100, 300 and 510) nm. Actual diameters reported on the calibration certificate were (80 ± 3, 100 ± 4, 303 ± 6 and 510 ± 7) nm. Aqueous PSS suspensions provided by the manufacturer were diluted in deionized water and atomized and dried using the same method as described in Section 2.1 of the main text. For SMPS calibrations, the dried PSS-laden aerosol plumes were drawn through the AAC without any aerodynamic size selection and the mobility size distribution was measured by the SMPS. The measured modal mobility size was compared to the actual diameters of the PSS as shown in Table S1. For both sizes the measured mobility size was within the range of sizes given by the manufacturers, therefore the SMPS did not require further calibration.

Actual PSS diameter / nm	Measured modal mobility diameter / nm
100 ± 4	102.4 ± 1.4
303 ± 6	302.3 ± 4.4

Table S1. Actual and measured mobility diameters of a selection on PSS used to validate the existing calibration of the SMPS. Note that the smallest and largest PSS sizes were obtained after the SMPS calibration had been validated and so are not included here. Measured mobility diameters are taken as the average of six repeat scans and the uncertainty is one standard deviation in this calculated mean.

For calibration of the AAC, the actual diameters of the PSS on the calibration certificates provided by the manufacturer were first converted to aerodynamic diameters using the particle mass density of 1.05 g cm^{-3} reported by the manufacturers, this value is inline with those reported by other authors (Pugh and Heuer 1957; Tabibian et al. 1956; Williams and Backus 1949). The Cunningham slip correction factor for the mobility diameter was calculated using Eqn. 6 in the main text using a mean free path value of 69.9 nm, this was the mean value measured by the SMPS across optical property measurements and corresponded to a pressure of ~99.5 kPa and temperature of ~27.6 ºC. The corresponding aerodynamic diameter for each mobility size was retrieved using a numerical convergence approach of Eqn. 5 from the main text. These aerodynamic diameters were (81.2, 101.5, 307.1 and 516.7) nm for the four sizes of PSS used. The AAC was operated in scanning mode and a CPC 3756 connected to the exhaust sampled at a flow rate of 0.3 L min^{-1}. This was a lower flow rate than used for optical property measurements and we have assumed the calibration factor to be flow rate independent. A continuous scan type was used, which ramped the speed of the classifier using a constant sheath flow to scan over the aerodynamic diameter range. For each sample, bidirectional scans were performed, with the classifier first scanning down in size (i.e. up in speed), before scanning up in size (down in speed). A sheath flow of 15 L min^{-1} and a total time of 600 s with a delay time of 13 s was found experimentally to provide best results. The delay time represents the time the particles take to pass from the exit of the classifier to the CPC and the time the CPC takes to respond. The value of the delay time was determined iteratively and corresponded to the time that provided the optimal overlap of the aerodynamic particle size distributions from the up scan and down scan, as recommended by the manufacturer. The delay time depends on aerodynamic particle size range, sample flow, type of CPC and the length of tubing between the AAC and SMPS, and it must therefore be determined experimentally for different experimental setups. Small differences between the calculated aerodynamic diameters for each PSS sample and the diameters measured by the AAC were observed. As a result, a multiplicative calibration factor was calculated to correct the aerodynamic diameters input to the AAC. A calibration factor for each scan in the bidirectional scan for each size of PSS was calculated by dividing the nominal d_{ae} of the PSS sample by the measured aerodynamic diameter. The measured aerodynamic size was quantified in two ways; the modal aerodynamic diameter from the AAC scan was used, and a lognormal distribution was also fitted to the data that provided the median aerodynamic diameter. Table S2 shows the calculated calibration factors for the four different PSS sizes using modal and median lognormal aerodynamic diameters. Calculated calibration factors ranged between 0.934 and 0.970 and showed no clear correlation with the size of the PSS samples. An average value of 0.954 ± 0.011 was calculated

across all PSS sizes, with the uncertainty representing one standard deviation in the calculated mean. This average multiplicative factor was applied to the aerodynamic diameters used during measurements to correct them for subsequent mass density calculations.

**PSS actual aerodynamic diameter / nm**	**Modal aerodynamic diameter / nm**	**Correction factor using modal diameter**	**Best fit median aerodynamic diameter / nm**	**Correction factor using best fit diameter**
81.2	86.6	0.938	85.4	0.951
101.5	105.7	0.961	106.1	0.956
307.8	322.6	0.952	324.1	0.948
516.7	534.9	0.966	540.1	0.957

Table S2. Measured aerodynamic diameters and corresponding calculated correction factor for the different size PSS. Note that for each sample the diameters have been averaged from the up and down scans and the total mean given above was calculated from the average across all scans.

S2. Cavity ring-down spectroscopy

Two CRDS channels operating at wavelengths of 405 and 658 nm were used in this work. Each optical cavity consisted of two highly reflective (R>99.997%) mirrors separated by a distance of 40 cm. This high reflectivity achieves long path lengths over which the laser light travels. The linewidth of the laser output was on the order of several GHz. The output power of continuous wave diode lasers (Toptica Photonics, iBeam Smart) was modulated using a digital square-wave drive function at a frequency of 2000 Hz and 50:50 duty cycle. Maximum laser powers specified by the manufacturer were 300 mW for the 405-nm laser and 130 mW for the 658-nm, although for this work the lasers were operated at 60% of their maximum output power. For the measurements in this work, each individual spectrometer was excited by its own dedicated laser.

For each laser pulse (with a duration of 250 μs) injected into the cavity, a fraction of light leaks out from the rear mirror. This was detected using a photomultiplier tube (PMT). The PMT gains were set to 50% of the maximum for both lasers. The voltage of the PMT was recorded by a 2.5 mega-samples per second (MS s^{-1}) data acquisition card (16-bit vertical resolution, 4 MS s^{-1} National Instruments PXIe-6124). During the 250 μs period while the laser output is low (during the OFF period of the duty cycle), the photodiode voltage is recorded and decays exponentially; this exponential decay in voltage is the ring-down trace. By fitting each acquired ring-down trace to a single exponential function (using the Discrete Fourier Transform algorithm described in Everest and Atkinson (2008)), the characteristic e^{-1} folding time (the ring-down time, τ) was determined. For each CRDS spectrometer, 2000 traces were recorded per second, with each of these traces used to obtain a value of τ, and the 2000 values were then averaged over the one second time period. Allan deviation analysis showed that our optical cavities were stable over time periods > 10 s and therefore average data over 1 s intervals was appropriate. Before and after each measurement on aerosol-laden samples, corresponding to an aerodynamic diameter set-point, a filtered sample devoid of aerosol particles was passed through the cavity to provide background measurements of the mean background ring-down time (τ_0) over a 60 s period. Typical mean τ_0 values were (17.1 ± 0.05) μs and (37.2 ± 0.55) μs for the 405-nm and 658-nm spectrometers, respectively. From measurements of τ_0 and the ring-down time in the presence of sample (τ), the extinction coefficient (α_{ext}) was calculated using Equation S2.1:

$$\alpha_{\text{ext}} = \frac{R_L}{c}\left(\frac{1}{\tau} - \frac{1}{\tau_0}\right) \qquad \text{S2.1}$$

in which c is the speed of light and R_L the ratio of the total cavity length (40 cm) to the length the sample occupies. This latter value was taken to be the distance between the sample inlet and outlet ports (34 cm), and therefore R_L took a value of 1.1765 for both CRDS channels used. Contamination of the mirror surface by aerosol deposition was mitigated against by using a small purge flow of zero air, with a total flow rate of 0.1 L min^{-1} from a mass flow controller that is split passively via a purge manifold to each

of the CRDS mirrors of the spectrometers used. This purge flow in the region close to the mirror surface prevented the aerosol sample from occupying the complete length of the cavity and the R_L factor accounts for this reduction in aerosol sample occupation over the optical path length. Uncertainty in the value of R_L can lead to bias in the measured extinction cross-sections. Our previously published analysis (see Table 1 of Cotterell et al. (2020)) indicates that an uncertainty in R_L of ~1.5% leads to a smaller systematic error in retrieved refractive indices for absorbing and non-absorbing aerosol particles in comparison to those uncertainties that arise from biases in, for example, the measured aerosol particle number concentration. The R_L factor is still an important source of bias and increased uncertainty in R_L above this 1.5% level would result in increasing bias in measured extinction cross-sections. Increased levels of noise in the ring-down times are observed when α_{ext} exceeds ~1000 Mm^{-1}. Typical ranges of α_{ext} for these measurements were < 350 Mm^{-1} and < 300 Mm^{-1} for the 405-nm and 658-nm spectrometers, respectively and consistently well below this level of increased noise. Calculations of α_{ext} included Rayleigh scattering and purge dilution factor corrections.

S3. Photoacoustic spectroscopy

Two photoacoustic spectrometers operating at optical wavelengths of 405 nm and 658 nm were used for absorption measurements. For each spectrometer, the output from a continuous wave diode laser (Toptica Photonics, iBeam Smart) was passed into an astigmatic multi-pass optical cavity. The laser beam was reflected multiple times (~50) through the photoacoustic cell using highly reflective cylindrical concave mirrors at either end of the cavity. The power of the laser was modulated sinusoidally at the resonant frequency of the photoacoustic cell. This cell adopted the two-resonator geometry reported in (Cotterell et al. 2019) and consisted of two half-wavelength resonators capped on each end with acoustic buffer volumes, with the laser passing through the lower resonator only. Brewster-angled windows were used to minimize detection of the laser interacting with the PAS cell windows to improve sensitivity. Small electret microphones were located at the center of each resonator, while a miniature speaker was located within one resonator only. The resonant frequency (or *eigenfrequency*) of the cell was determined by emitting a 1-second 'chirp' from the speaker spanning a range of frequencies ± 200 Hz around the expected resonant frequency. A Fourier transform of the microphone response following this chirp provided an acoustic spectrum characterizing the excitation efficiency of the cell at different frequencies, and the maximum in this spectrum corresponded to the resonant frequency.

Light absorbing aerosols in the sample are heated when irradiated by the laser. This energy is then transferred to translational modes (kinetic energy) of surrounding bath gas molecules. The bath gas undergoes adiabatic expansion causing an increase in pressure. Since the laser power is modulated sinusoidally, pressure (acoustic) waves are generated. If the laser is modulated at the same resonant frequency of the cell, amplification of the sound waves occurs by their coupling into the resonant eigenmode of the photoacoustic cell. This amplified sound wave was detected by sensitive microphones in each resonator center, and a differential detection scheme was applied to these microphone responses (see Cotterell et al. (2019)). The amplitude of this microphone response was related to the absorption coefficient via a calibration, performed using a sample with known absorption. Calibration of the photoacoustic spectrometers was achieved using ozone gas as a calibrant. The absorption can be determined by simultaneously measuring the extinction using CRDS. The validity of ozone as a calibrant for PAS was tested by Davies et al. (2018), and excellent agreement was found between measured and modelled absorption cross-sections for light absorbing aerosols, when applying ozone calibrations, at wavelengths of (405, 514 and 658) nm.

S4. CPC transmission factors

Transmission factors were measured by atomizing and drying samples of ammonium sulfate or nigrosin and size selecting with the AAC at the extreme aerodynamic diameters used in measurements (100 nm and 400 nm). The number concentration at the exit port of the 405-nm PAS was measured for five minutes by the CPC 3789 before it was moved to the 658-nm PAS exit port, this process was repeated

to give ten minutes of number concentration measurements for each sampling line. For the spectrometer not attached to the CPC, a flow of 0.6 L min^{-1} was drawn through the spectrometer using a vacuum pump connected and regulated using a mass flow controller. There were considerable differences in number concentrations recorded between the two lines and a transmission factor (T_{658}) to correct measured concentrations from the 405-nm PAS cell to a value representative of the 658-nm PAS cell was calculated using equation S4.1 in which N_{658} and N_{405} are the average number concentrations measured from the 658-nm PAS cell and the 405-nm PAS cell respectively.

$$T_{658} = \frac{N_{658}}{N_{405}} \qquad \text{S4.1}$$

There was no clear size dependency to the transmission factor and a mean transmission factor across ammonium sulfate and nigrosin aerosols at 100 nm and 400 nm aerodynamic diameter was calculated to be $T_{658} = 1.243 \pm 0.019$. The aerosol number concentrations in the 658-nm channel were ~24% larger than those in the 405-nm channel. Consequently, the measured number concentrations were corrected using the transmission factor during the data analysis process.

S5. Example SMPS data

Example data obtained from the SMPS at each selected aerodynamic diameter is shown in Figure S1, the result of fitting a bimodal lognormal distribution is also shown for each scan. Multiple peaks are observed in these mobility size distributions (inferred from the measured electrical mobility distribution), particularly at higher aerodynamic size selection. The origin of these peaks can be attributed to the Boltzmann-like charge distribution applied by the x-ray neutralizer. This charge distribution results in most particles being imparted with a single unit charge and some gaining additional charges (Tigges et al. 2015a, 2015b). The highest intensity peak in the mobility size distribution corresponds to the singly charged (q = 1) particles while further peaks correspond to particles with increasing charge (q = 2, 3 etc.). Prior to the charging process, a monodisperse distribution of aerodynamic particle sizes is obtained using the AAC. As a result, these additional peaks have the same geometric size as the q = 1 peak but a higher charge, so they appear at smaller mobility size. This is because the mobility of a particle is proportional to the charge and inversely proportional to the diameter (Stolzenburg and McMurry 2008).

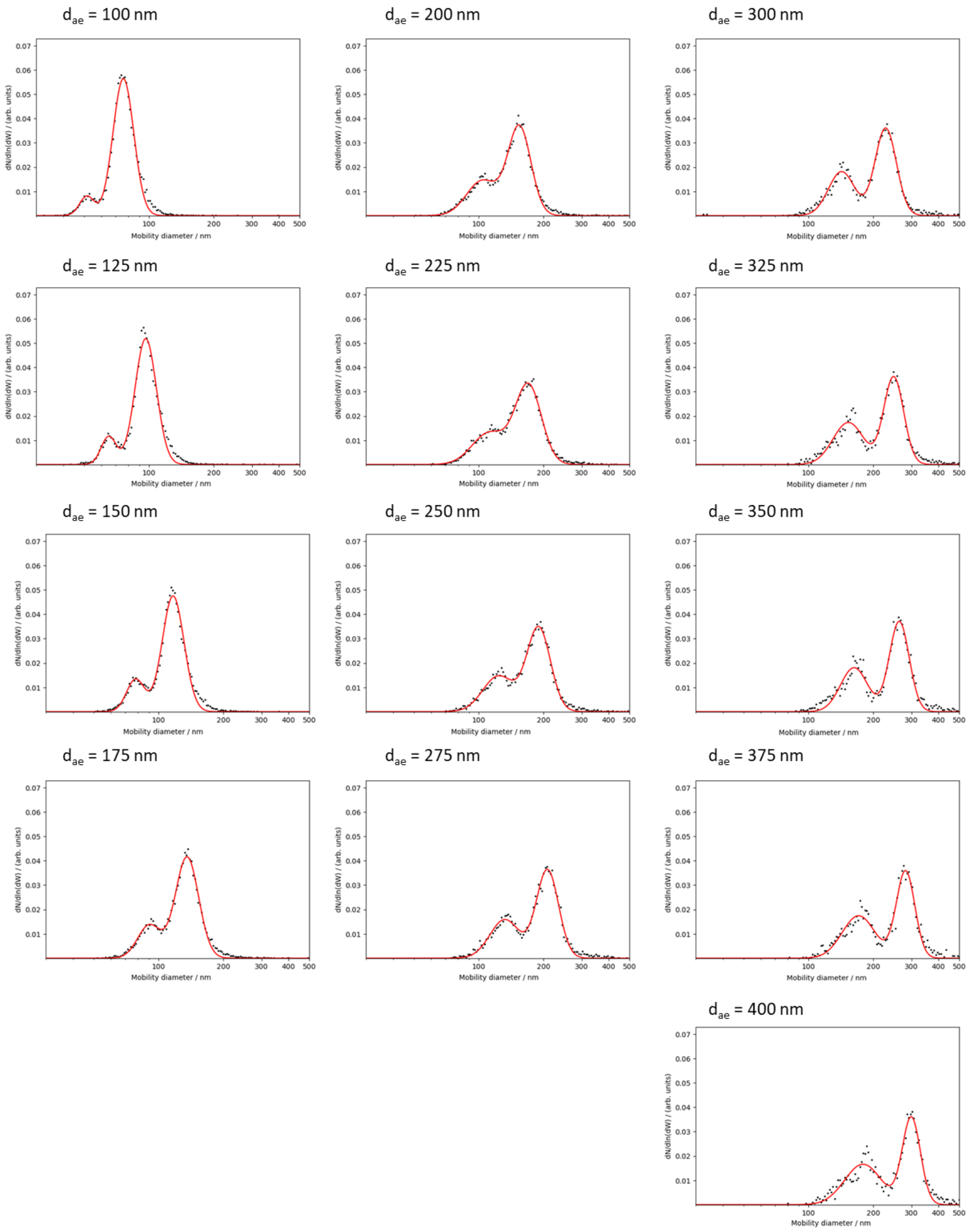

Figure S1. Example mobility size distributions measured by the SMPS for each selected aerodynamic diameter for nigrosin aerosol particles. The black points are the normalized measured concentrations, and the red lines are the best fit bimodal lognormal distributions to the measured data.

S6. Expanded scales for measured and modelled cross-section plots

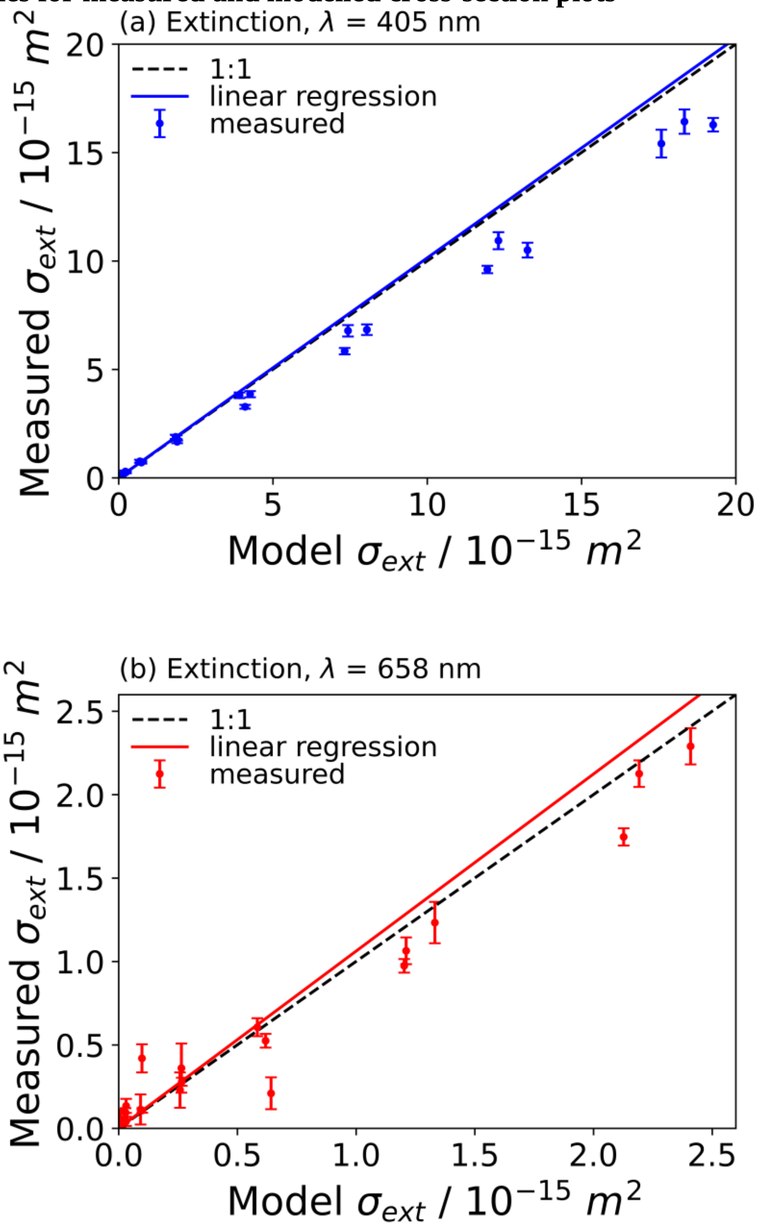

Figure S2. Expanded scale of the data from Figure 2 of the main text to show the measured and modelled extinction cross-sections for ammonium sulfate at lower magnitudes. The ordinary least squares linear regression from Figure 2, and a 1:1 line are shown for comparison.

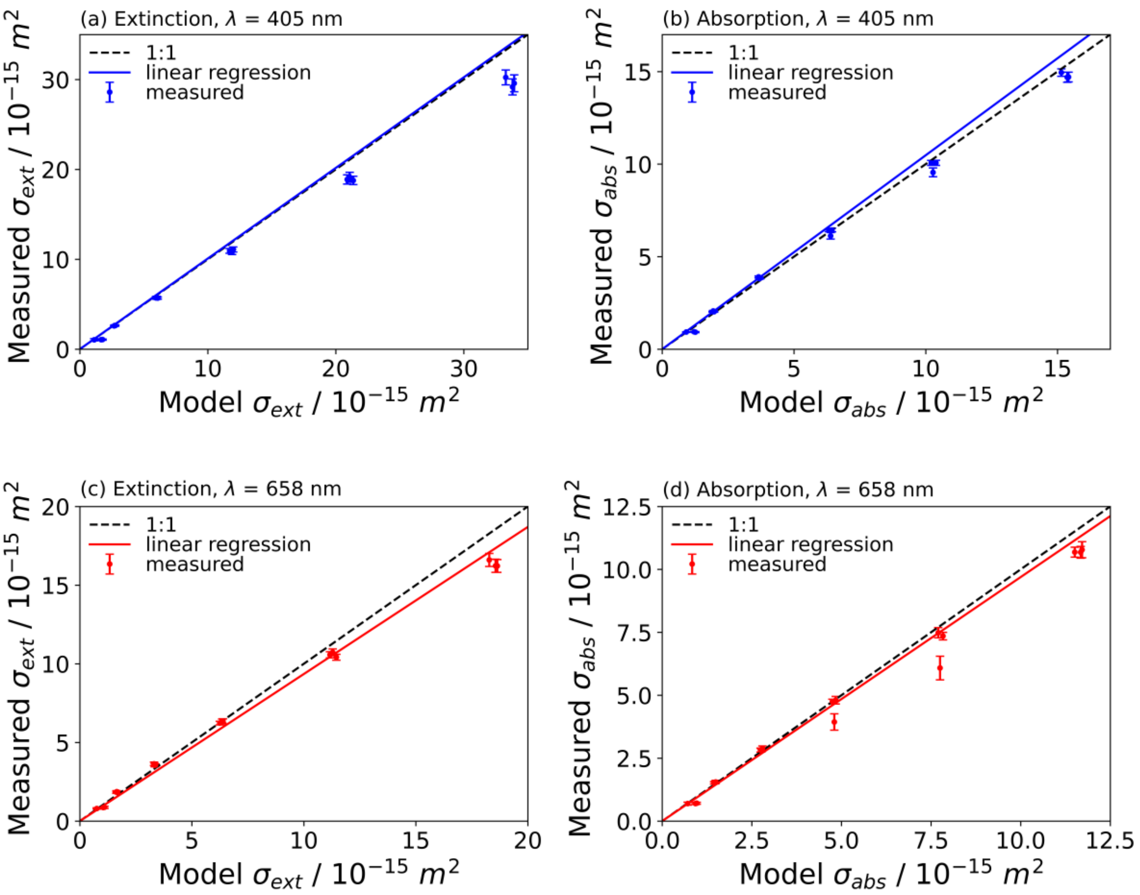

Figure S3. Expanded scale of the data from Figure 4 of the main text to show the measured and modelled extinction and absorption cross-sections for nigrosin at lower magnitudes. The ordinary least squares linear regression from Figure 4, and a 1:1 line are shown for comparison.

S7. Residual plots for ordinary least-squares linear regressions

Residual plots to show the differences between measured values and those predicted by the OLS linear regression used in the main text are given in Figure S4. For the 405-nm extinction data the residuals seem to be low biased at the lower magnitudes of extinction cross-section. This shows that the OLS linear regression consistently overestimates the value of extinction cross-section in this region. This observation may show that a better representation of the data would be to use a weighted least squares regression to better capture this data as discussed in Section S8.

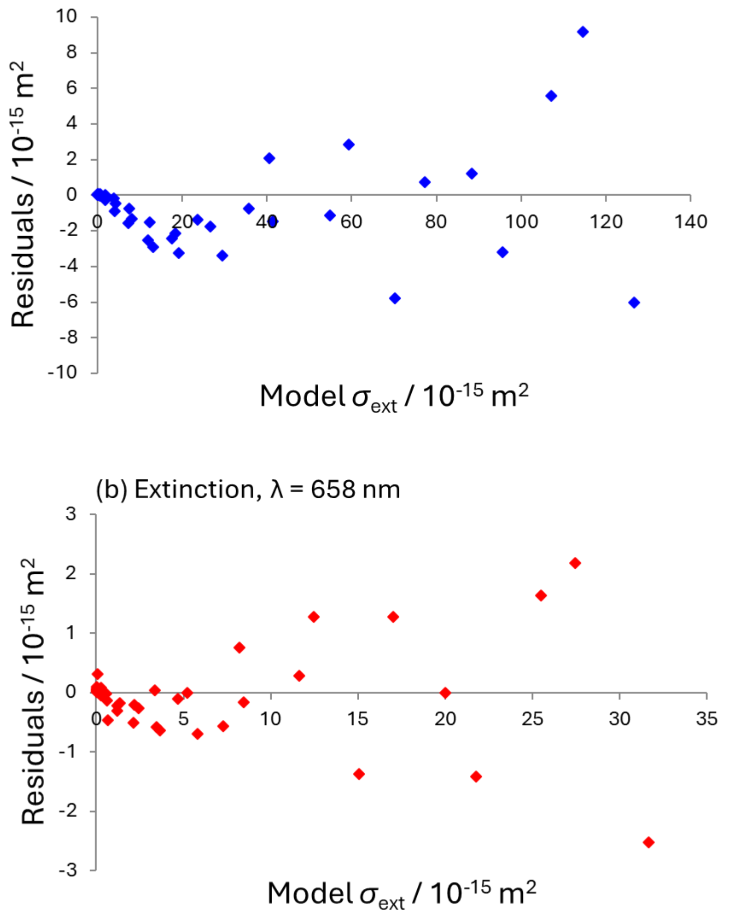

Figure S4. Residual plots for ordinary least-squares linear regression for ammonium sulfate extinction cross-section datasets from Figure 2 of the main text, measured at (a) 405 nm and (b) 658 nm. Residuals are calculated by subtracting the value predicted by the OLS linear regression from the measured value.

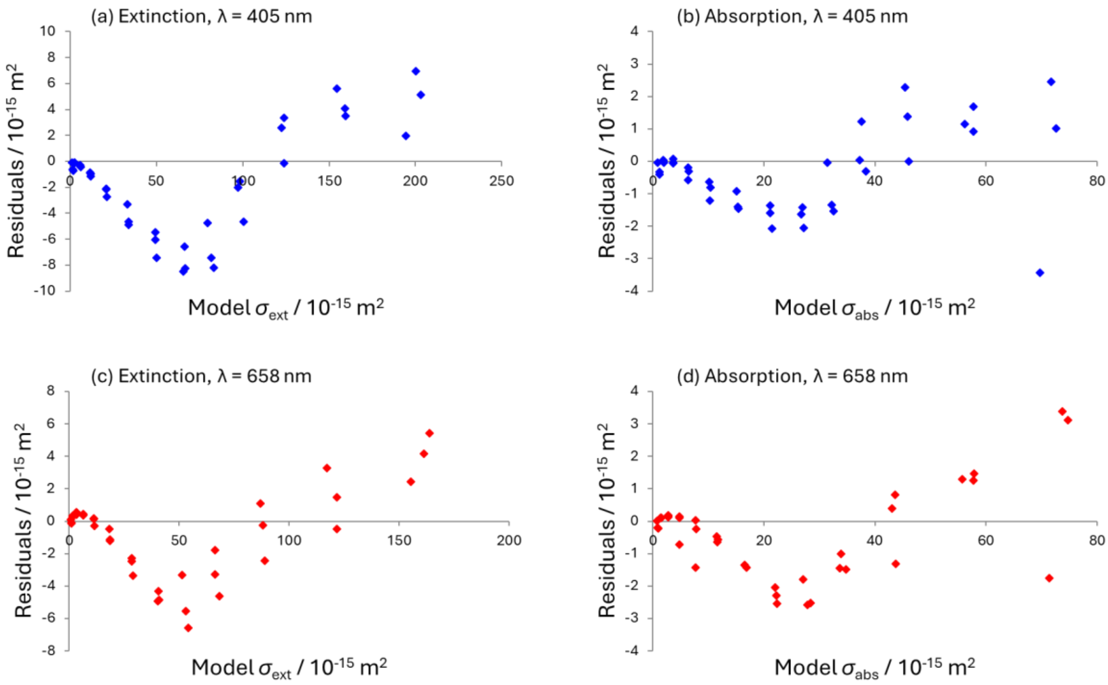

Figure S5. Residual plots for ordinary least-squares linear regressions for nigrosin optical cross-section datasets from Figure 4 of the main text. Extinction cross-sections are measured at (a) 405 nm, and (c) 658 nm and absorption cross-sections are measured at (b) 405 nm, and (d) 658 nm.

S8. Comparison of precision in cross-section measurements using mobility and aerodynamic size selection

The precision of our AAC selected particles is compared to that of mobility selected particles measured by Cotterell et al. (2020) using the same optical spectrometers. Extinction cross-sections for ammonium sulfate aerosols and their associated standard deviations are shown in Figure S6. Extinction cross-sections in the same range of model values are shown and larger cross-sections measured by Cotterell et al. (2020) are excluded from the comparison since standard deviation scales with the magnitude of the measured cross-section. The error bars for aerodynamically selected aerosols are considerably smaller than those for mobility selected aerosol for both optical wavelengths used. This increased precision when using an AAC can be attributed to the higher transmission efficiency resulting in larger number concentration as well as the removal of multiply charged particles present with a DMA which do not need to be corrected for.

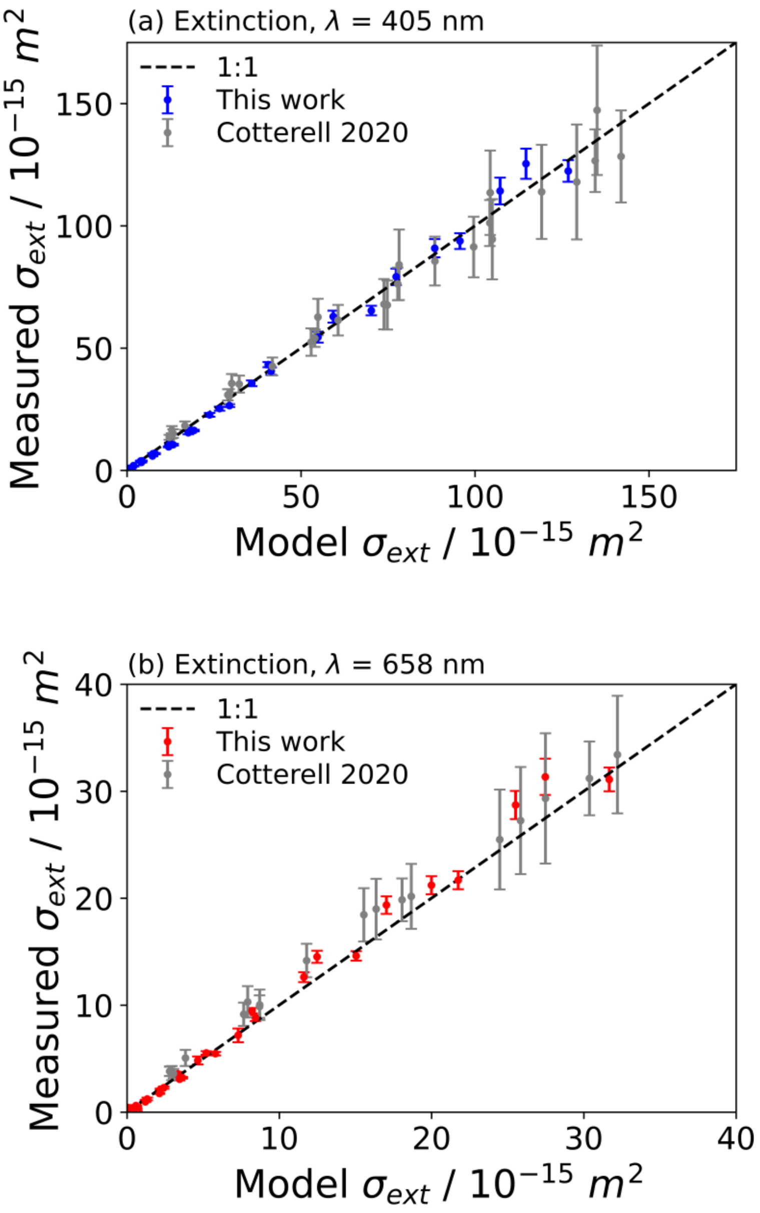

Figure S6. Comparison of measured and modelled cross-sections for ammonium sulfate aerosols selected by aerodynamic (red and blue, this work) and mobility (grey, Cotterell et al. (2020)) diameters, measured at optical wavelengths of (a) 405 nm and (b) 658 nm. Error bars represent one standard deviation in the measured cross-sections.

The precision of extinction and absorption cross-sections of our aerodynamically selected nigrosin aerosol is compared to mobility selected measurements made by Cotterell et al. (2020) in Figure S7. The same trend of increased precision for the measurements made with the AAC is seen when compared to similar values measured using a DMA for both optical wavelengths. The observed precision is especially improved for the absorption cross-section measurements as these show a greater decrease in uncertainty, particularly at lower values of absorption cross-section and for the measurements made at 658 nm.

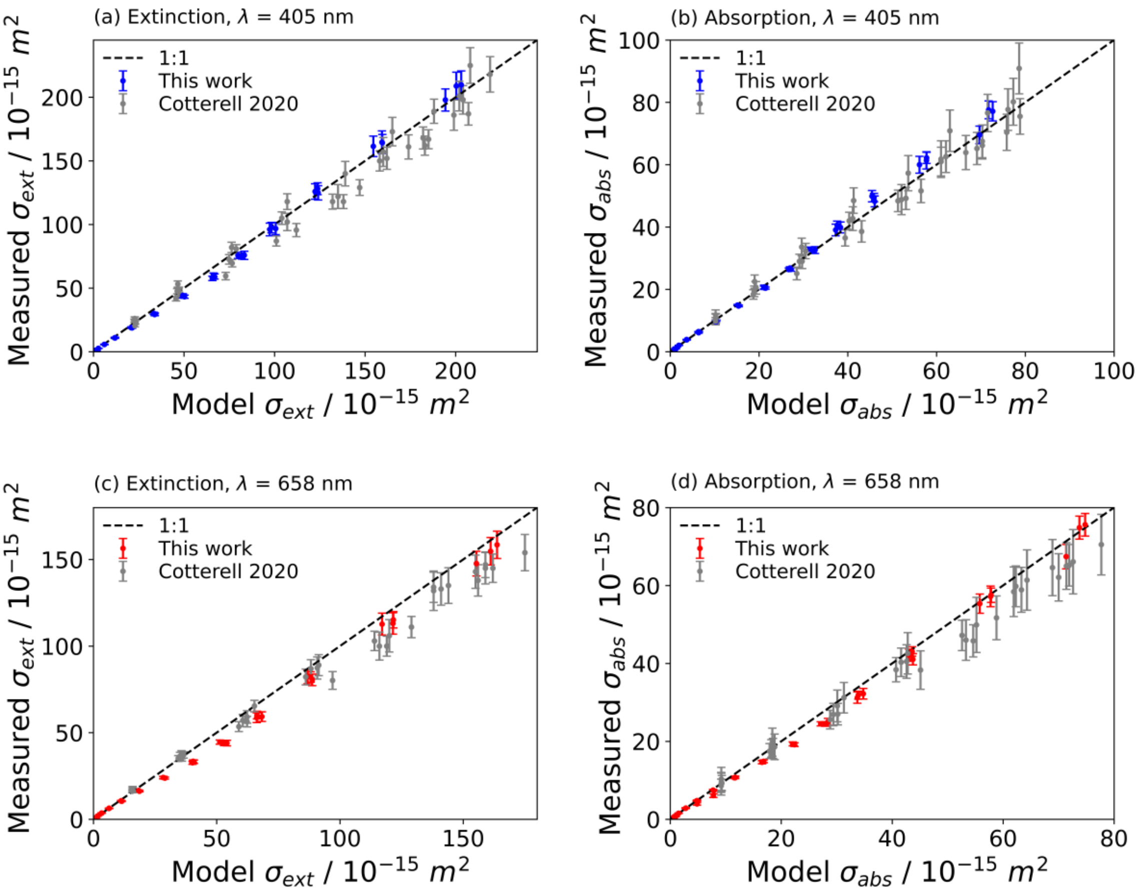

Figure S7. Comparison of measured and model extinction cross-sections for aerodynamic (red and blue, this work) and mobility (grey, Cotterell et al. (2020)) selected nigrosin aerosol particles at optical wavelengths of (a) 405 nm and (c) 658 nm. Comparison of measured and model absorption cross-sections for aerodynamic (red, this work) and mobility (grey, Cotterell et al. (2020)) selected nigrosin aerosol particles at optical wavelengths of (b) 405 nm and (d) 658 nm. Error bars represent one standard deviation in measured cross-sections.

S9. Sucrose optical cross-sections

The measured extinction cross-sections of aerosols comprised of sucrose as a function of mobility diameter are shown in Figure S8. The lack of reported literature values for refractive indices of dry, pure sucrose aerosols means a comparison with modelled cross-sections is not possible. Figure S9 shows the measured absorption coefficients for non-absorbing sucrose aerosols. These values all lie at zero within one standard deviation as expected for a non-absorbing aerosol.

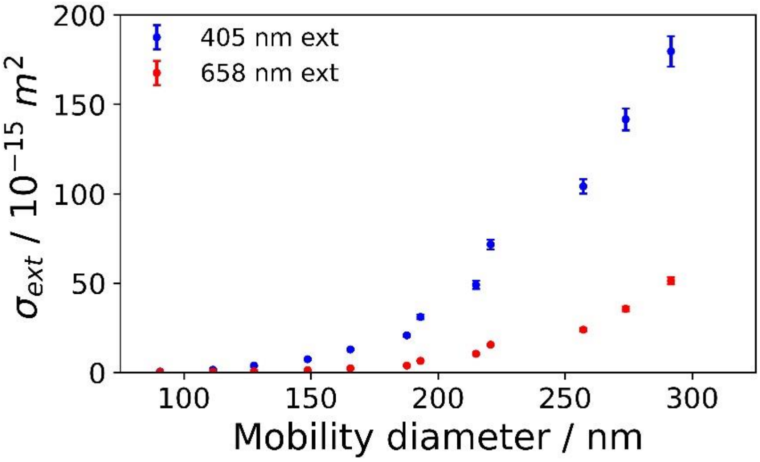

Figure S8. Measured extinction cross-sections for a representative data set of pure sucrose aerosols measured at 405 nm and 658 nm. Error bars represent one standard deviation in measurements.

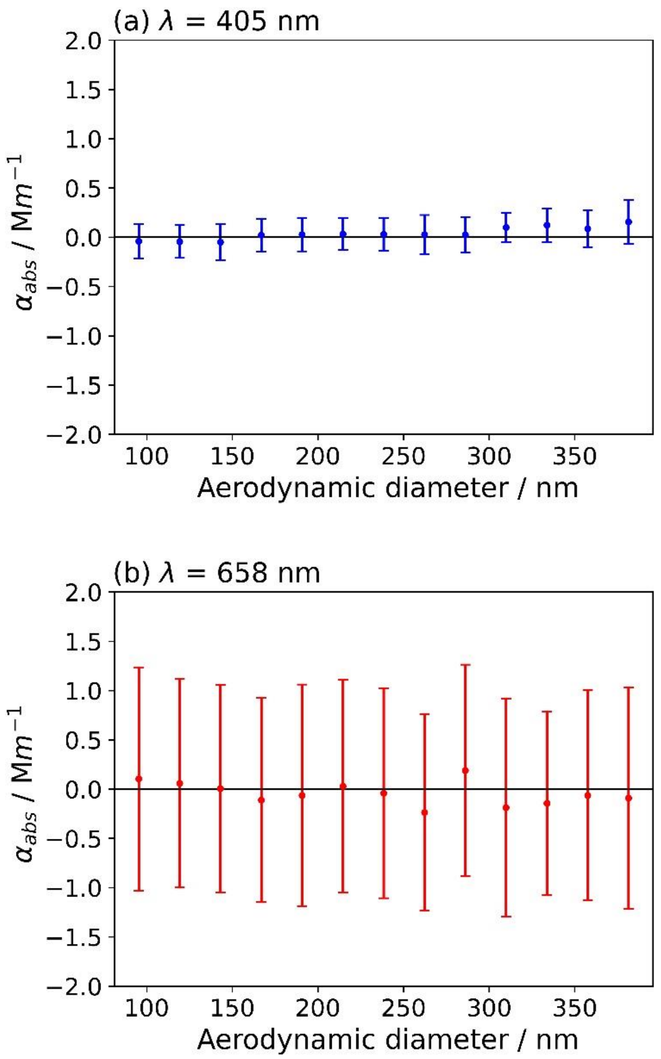

Figure S9. Measured absorption coefficients at each selected aerodynamic diameter for one repeat measurement of aerosols consisting of pure sucrose measured at (a) 405 nm and (b) 658 nm. Error bars show the variation in values across the five-minute sampling period.

S10. Comparison of ordinary and weighted least-squares linear regressions

Linear regressions from Figure 2 of the main text were repeated using a weighted least-squares procedure to fit a straight line to the data forced through the origin. The original ordinary least-squares (OLS) and the weighted least-squares (WLS) for ammonium sulphate are shown in Figure S10. Linear regressions were also repeated with weightings to account for uncertainty in measurements for the nigrosin data shown in Figure 3 of the main text, the OLS and WLS linear regressions are compared in Figure S11. The least-squares procedure was weighted for measurement uncertainty by weighting by the inverse of the variance. The choice of weighting can impact the slope of the linear regression performed between measured and modelled extinction cross-sections as seen in Figure S10 for ammonium sulfate. The WLS regression biases the slope towards the extinction cross-sections with lower standard deviations which effectively biases it to the smallest magnitude cross-sections.

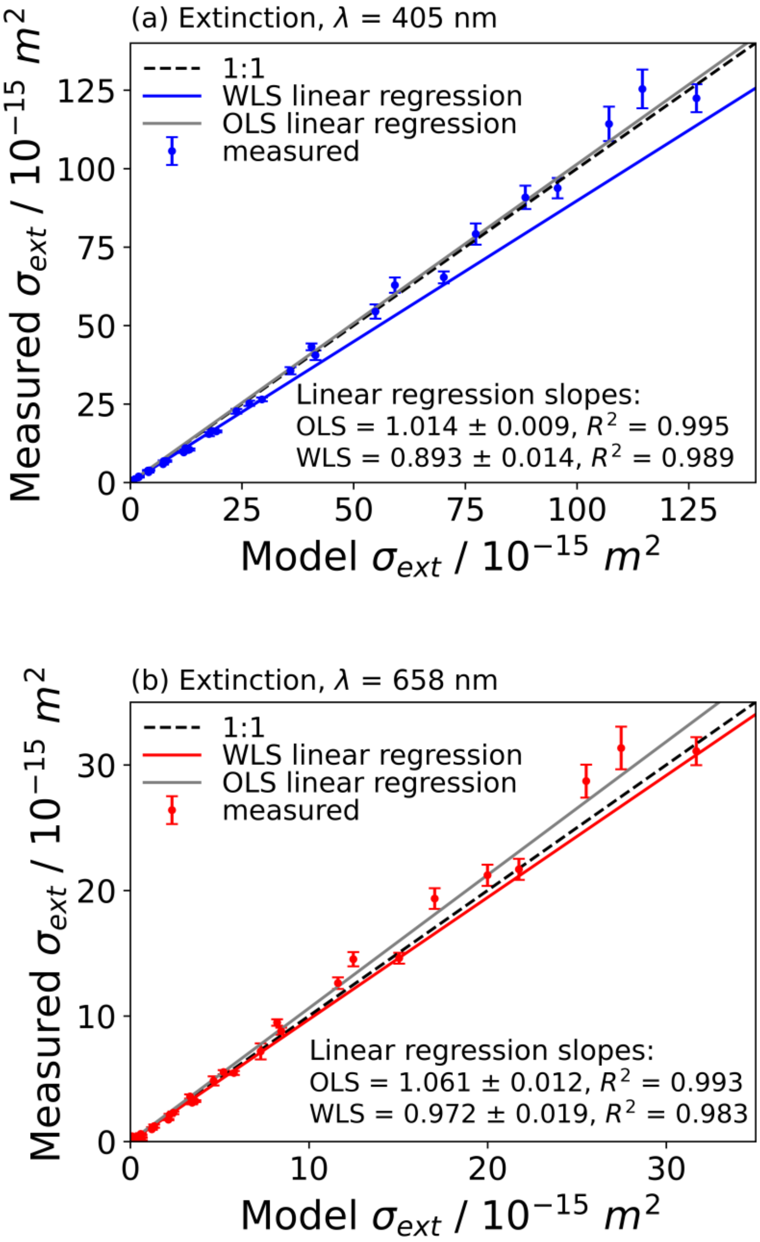

Figure S10. Measured and model extinction cross-sections for aerodynamically selected ammonium sulfate aerosols particles at optical wavelengths of (a) 405 nm, and (b) 658 nm. The original ordinary least squares (OLS) linear regressions from Figure 2 are shown in grey and compared to weighted least-

squares (WLS) linear regressions. WLS linear regressions were performed by weighting by the inverse of the variance of the measurements. The values for linear regression slopes, their associated standard error and coefficient of determination are given for both linear regression approaches. Again, particularly for the 405-nm extinction data, the choice of weighting affects the value of the linear regression slope between measured and modelled values. It is possible that values at lower magnitude extinction cross-sections are low-biased which results in the weighted linear regression having a lower slope than that for the ordinary regression.

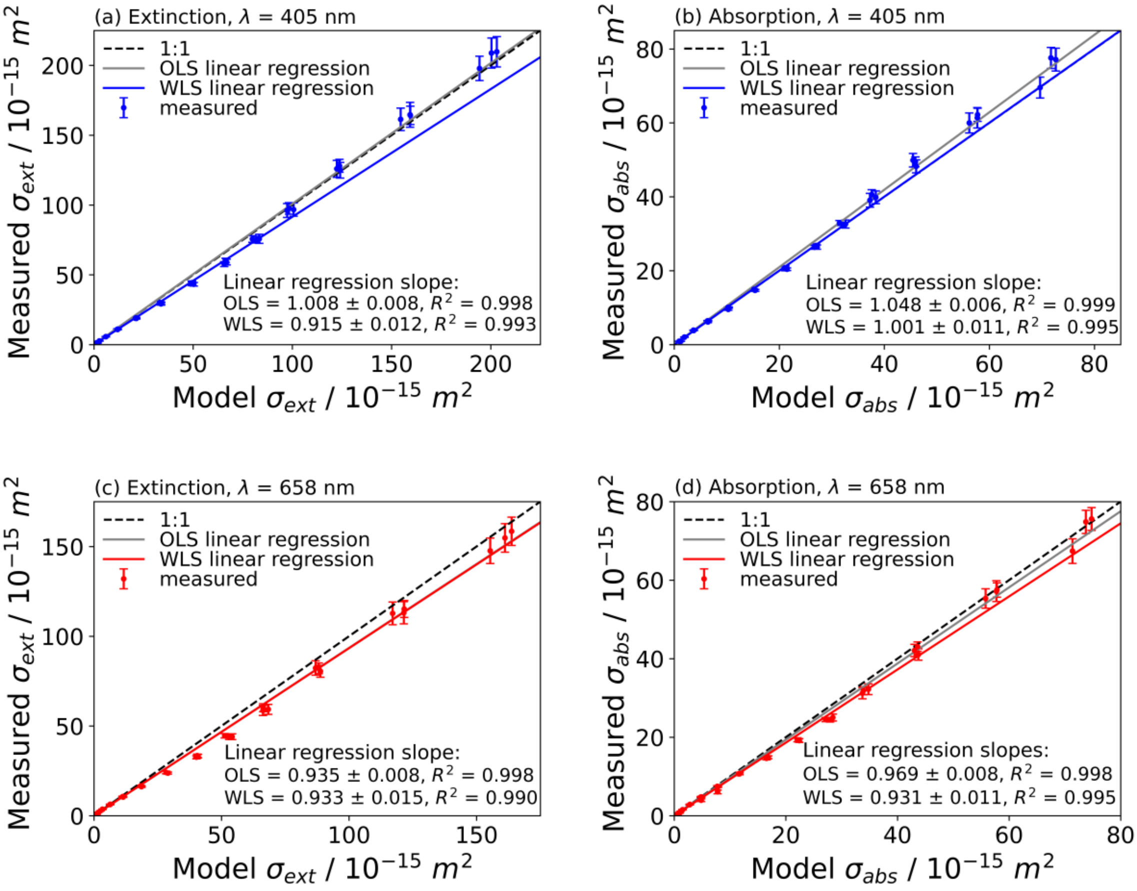

Figure S11. Measured and model optical cross-sections for aerodynamically selected nigrosin aerosol particles. Extinction cross-sections measured at optical wavelengths of (a) 405 nm and (c) 658 nm and absorption cross-sections measured at optical wavelengths of (b) 405 nm and (d) 658 nm. The original ordinary least squares (OLS) linear regressions from Figure 3 are shown in grey and compared to weighted least-squares (WLS) linear regressions. WLS linear regressions were performed by weighting by the inverse of the variance of the measurements. The values for linear regression slopes, their associated standard error and coefficient of determination are given for both linear regression approaches.

Ordinary least-squares linear regressions were repeated allowing the intercepts to vary and not forced through the origin. P-values were calculated for these intercepts with a null hypothesis of intercepts being equal to zero. These calculated intercepts and their associated p-values are given in Table S3 for all pure component cross-section measurements.

**Species**	**Wavelength / nm**	**Cross-section**	**Intercept / 10^{-15} m^2**	***p-value***
ammonium sulfate	405	extinction	-0.973 ± 0.535	0.0774
ammonium sulfate	658	extinction	-0.088 ± 0.160	0.5811
nigrosin	405	extinction	-3.498 ± 0.853	0.0002
nigrosin	658	extinction	-1.560 ± 0.560	0.0083
nigrosin	405	absorption	-0.784 ± 0.294	0.0114
nigrosin	658	absorption	-0.899 ± 0.307	0.0058

Table S3. Summary of intercept values for repeating an ordinary least squares regression of the cross-section datasets allowing the intercept to vary. P-values calculated with a null hypothesis that the intercept is zero. P-values of > 0.05 represent to a 95% confidence interval that there is no statistically significant difference between the intercept and zero.

S11. Absorption coefficients of ammonium sulfate

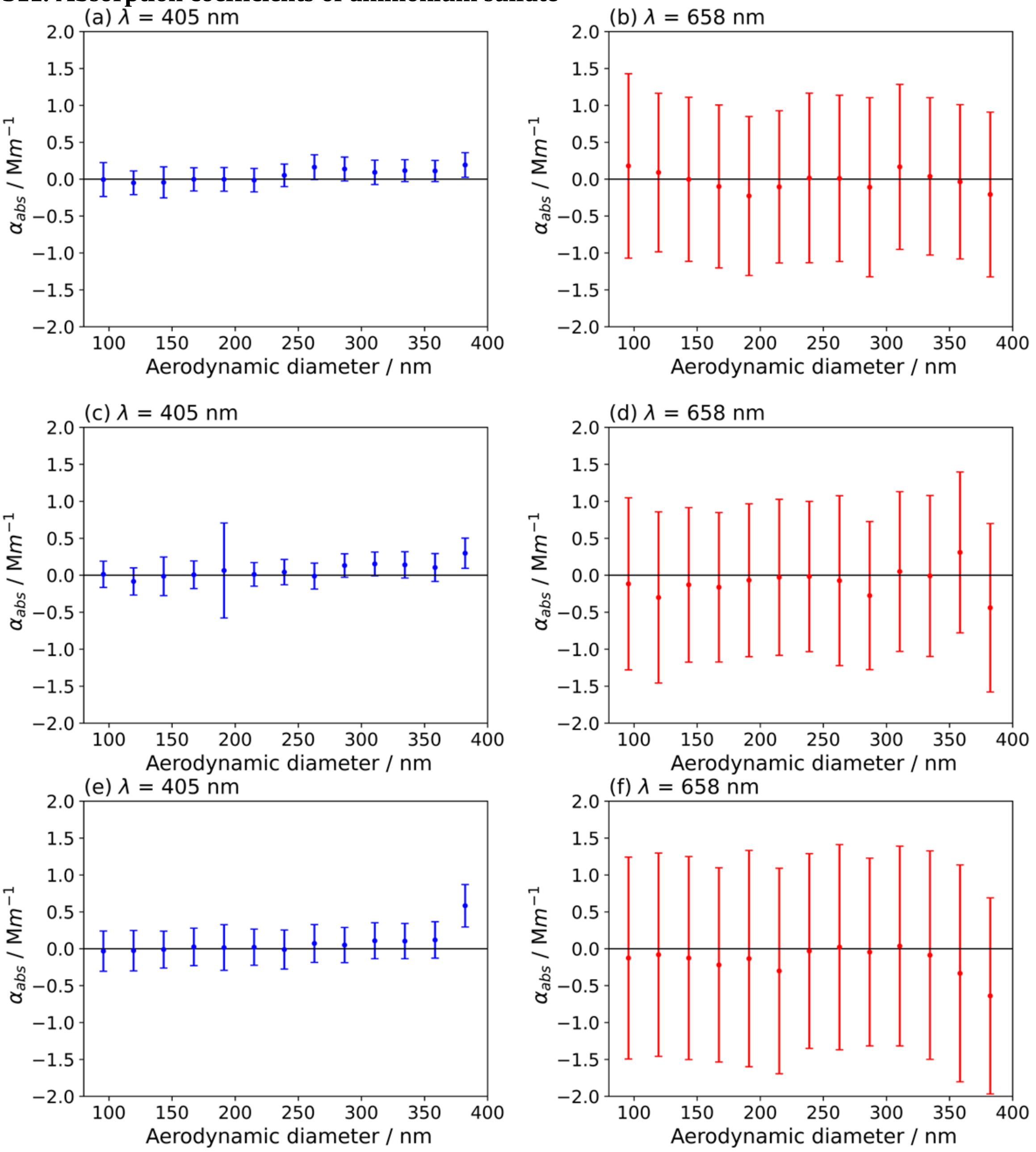

Figure S12. Measured absorption coefficients at each selected aerodynamic diameter for three repeat measurements of pure ammonium sulfate aerosol particles measured at wavelengths of (a, c, e) 405 nm or (b, d, f) 658 nm. Error bars show the standard deviation in values across the five-minute sampling period. Aerodynamic diameters are corrected from input values using the measured calibration factor.

S12. Effective mass density characterizations for a range of two-component mixtures

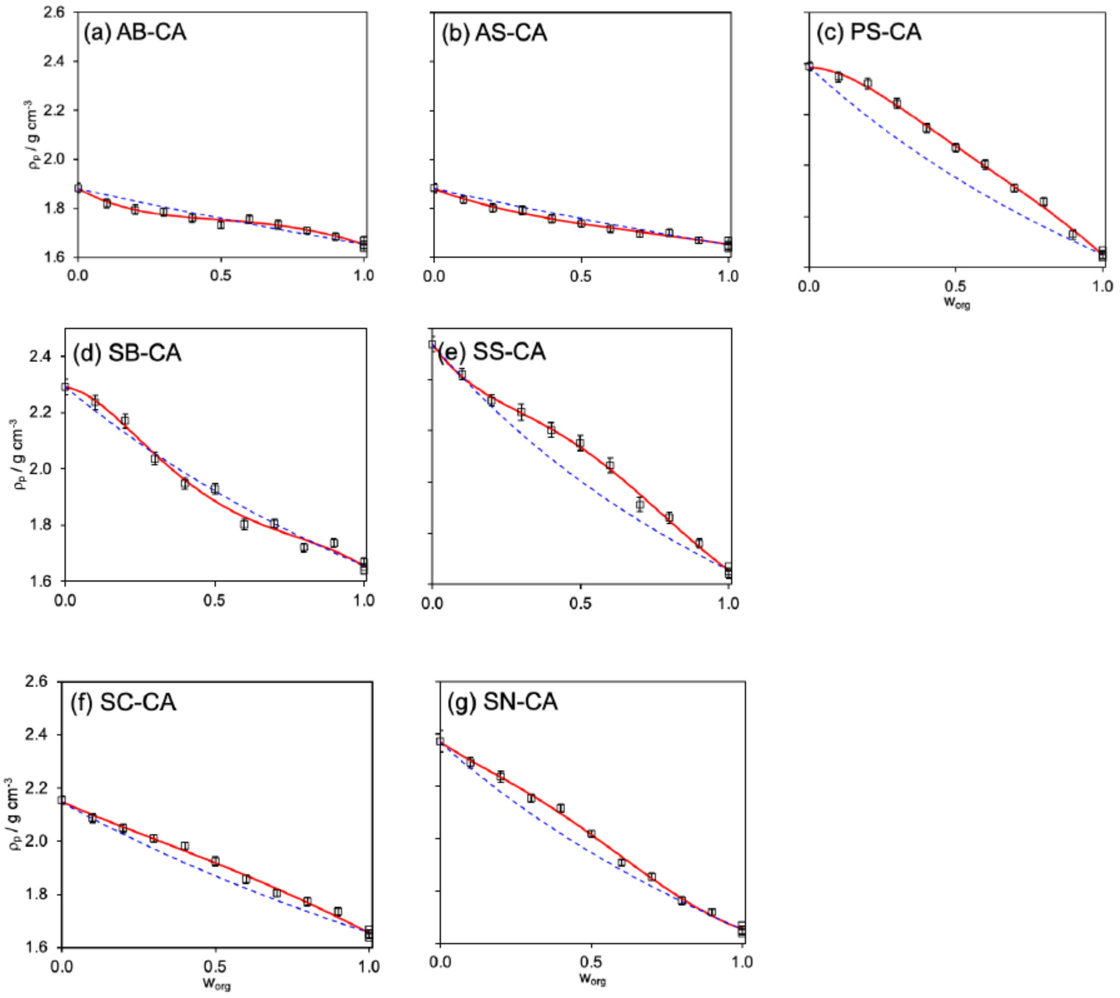

Figure S13. Effective particle mass density (ρ_p) versus organic mass fractions (w_{org}) for internally mixed, two-component organic-inorganic aerosol particles containing the organic species citric acid (CA). The inorganic species explored include ammonium bisulfate (AB), ammonium sulfate (AS), potassium sulfate (PS), sodium bisulfate (SB), sodium sulfate (SS), sodium chloride (SC), and sodium nitrate (SN). The black squares show the measured particle densities. The error bars indicate the standard error in the linear regression used to determine mass density. The red lines represent the best fits of a polynomial model, and the blue dashed lines represent the ideal mixing model.

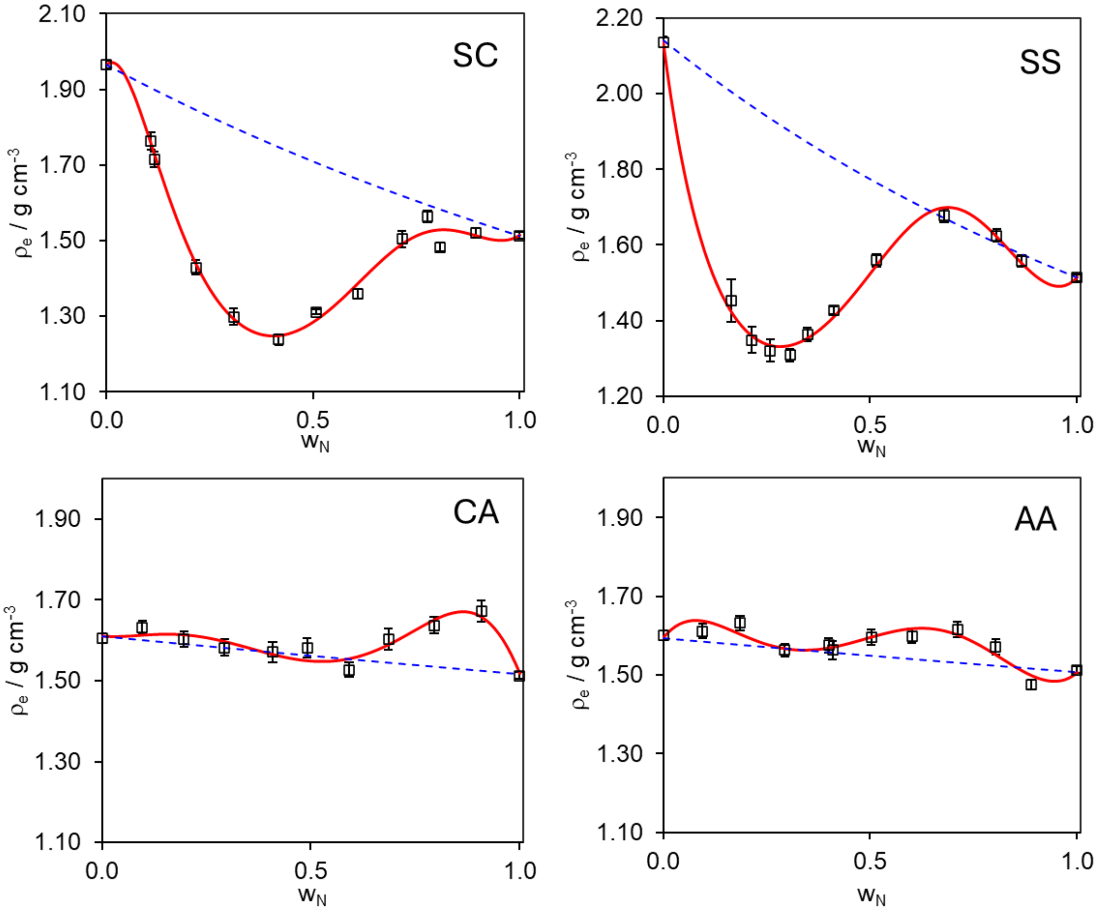

Figure S14. Particle effective mass density (ρ_e) versus nigrosin mass fraction (w_N) for internally mixed, two-component aerosol particles containing nigrosin mixed with a further species. This secondary species corresponds to sodium chloride (SC), sodium sulfate (SS), citric acid (CA), or ascorbic acid (AA) as indicated in the panel labels. The black squares show the measured particle densities. The error bars indicate the standard error in the linear regression used to determine mass density. The red lines represent the best fits of a polynomial model, and the blue dashed lines represent the ideal mixing model.

S13. Further examination of the molar refraction mixing rule

We renew the sentiments of Liu and Daum (2008) in stressing the importance of using refractive index mixing models that have an underlying physical basis that arise from the dependence of the refractive index on molar polarizability and extend this argument to light absorbing aerosol particles that have received little attention in the literature with respect to refractive index prediction from appropriate mixing models. Mayerhöfer and Popp (2020) derive the molar refraction mixing rule in terms of the complex refractive index, and it can be shown that the molar refraction (R) of a substance is given by:

$$R = \left(\frac{m^2 - 1}{m^2 + 2}\right)\frac{M}{\rho} = r\frac{M}{\rho} = \frac{N_A\hat{\alpha}}{3} \qquad \text{S12.1}$$

in which m is the complex refractive index, M is the molecular weight, ρ is the mass density, r is the specific refraction, N_A is the Avogadro constant, and $\hat{\alpha}$ is the complex molecular polarizability. The mole fraction weighting of molar refraction (molar refraction mixing rule) applied to a two-component mixture of species A and B with respective mole fractions x_A and x_B is then:

$$R_e = x_A R_A + (1 - x_A)R_B \qquad \text{S12.2}$$

in which R_e is the effective molar refraction of the mixture, and R_A and R_B are the molar refractions of pure A and B. We can combine the last two equations to write:

$$\left(\frac{m_e^2-1}{m_e^2+2}\right)\frac{M_e}{\rho_e}=x_A\left(\frac{m_A^2-1}{m_A^2+2}\right)\frac{M_A}{\rho_A}+(1-x_A)\left(\frac{m_B^2-1}{m_B^2+2}\right)\frac{M_B}{\rho_B} \qquad \text{S12.3}$$

This equation shows that the complex refractive index of the mixture (m_e) can be calculated from those of the pure components (m_A and m_B) provided the molecular weights and densities of the pure components and the mixture are known. The molecular weights and densities of pure materials are usually known, while the effective molecular weight is calculated from the application of mass conservation and is given simply by:

$$M_e = x_A M_A + (1-x_A)M_B \qquad \text{S12.4}$$

As described in the main manuscript, the effective mass density is often treated using the ideal mixing rule for mass density, but predictions of refractive indices for mixtures are better captured if accurate descriptions of the mixture mass density are available from measurement data.

There are two problems with the study of Cotterell et al. (2020) on the refractive index of mixtures. First, the molar refraction mixing rule was applied to the prediction of the real component of the mixture refractive index (n_e) by replacing the m terms in Eqn. S12.3 with the real component n only. That is, the impact of light absorption (and therefore a non-negligible k) on the predicted n_e was ignored; as we show below, this simple replacement is not strictly allowed. Second, different mixing rules were used to predict n_e and k_e, with the latter predicted using a linear mass fraction weighting of the k for the pure species. Yet, the molar refraction mixing framework above (Eqn. S12.3) includes explicitly the complex refractive index and therefore should apply to both the real and imaginary components. This previous separation in treatments for n and k is not justified; both components should be predicted from the same effective medium approximation framework and, as we show below, n and k are mutually dependent.

We can continue to develop Eqn. S12.3 by writing the complex refractive index terms in terms of their real and imaginary components:

$$\begin{aligned}R_e &= x_A\left(\frac{(n_A+ik_A)^2-1}{(n_A+ik_A)^2+2}\right)\frac{M_A}{\rho_A}+(1-x_A)\left(\frac{(n_B+ik_B)^2-1}{(n_B+ik_B)^2+2}\right)\frac{M_B}{\rho_B}\\ &= x_A\left(\frac{{n_A}^2-{k_A}^2+i2n_Ak_A-1}{{n_A}^2-{k_A}^2+i2n_Ak_A+2}\right)\frac{M_A}{\rho_A}+(1-x_A)\left(\frac{{n_B}^2-{k_B}^2+i2n_Bk_B-1}{{n_B}^2-{k_B}^2+i2n_Bk_B+2}\right)\frac{M_B}{\rho_B}\\ &= \frac{x_AM_A}{\rho_A}r_A+\frac{(1-x_A)M_B}{\rho_B}r_B\end{aligned} \qquad \text{S12.5}$$

Looking at the specific refraction (r_i) terms in the last expression of the above equality more closely, we can remove the imaginary components from the denominator such that imaginary terms appear in the numerator only:

$$\begin{aligned}r_i &= \frac{{n_i}^2-{k_i}^2+i2n_ik_i-1}{{n_i}^2-{k_i}^2+i2n_ik_i+2}\\ &= \frac{\left({n_i}^2-{k_i}^2+i2n_ik_i-1\right)\left({n_i}^2-{k_i}^2-i2n_ik_i+2\right)}{\left({n_i}^2-{k_i}^2+i2n_ik_i+2\right)\left({n_i}^2-{k_i}^2-i2n_ik_i+2\right)}\\ &= \frac{n_i^4+k_i^4+n_i^2-k_i^2+2n_i^2k_i^2-2+i6n_ik_i}{n_i^4+k_i^4+4n_i^2-4k_i^2+2n_i^2k_i^2+4}\\ &= \frac{n_i^4+k_i^4+n_i^2-k_i^2+2n_i^2k_i^2-2}{n_i^4+k_i^4+4n_i^2-4k_i^2+2n_i^2k_i^2+4}+i\frac{6n_ik_i}{n_i^4+k_i^4+4n_i^2-4k_i^2+2n_i^2k_i^2+4}\end{aligned} \qquad \text{S12.6}$$

Substituting Eqn. S12.6 into Eqn. S12.5:

$$R_\mathrm{e} = \frac{x_\mathrm{A} M_\mathrm{A}}{\rho_\mathrm{A}} \left(\frac{n_\mathrm{A}^4 + k_\mathrm{A}^4 + n_\mathrm{A}^2 - k_\mathrm{A}^2 + 2n_\mathrm{A}^2 k_\mathrm{A}^2 - 2}{n_\mathrm{A}^4 + k_\mathrm{A}^4 + 4n_\mathrm{A}^2 - 4k_\mathrm{A}^2 + 2n_\mathrm{A}^2 k_\mathrm{A}^2 + 4} + i \frac{6 n_\mathrm{A} k_\mathrm{A}}{n_\mathrm{A}^4 + k_\mathrm{A}^4 + 4n_\mathrm{A}^2 - 4k_\mathrm{A}^2 + 2n_\mathrm{A}^2 k_\mathrm{A}^2 + 4} \right) + \frac{(1 - x_\mathrm{A}) M_\mathrm{B}}{\rho_\mathrm{B}} \left(\frac{n_\mathrm{B}^4 + k_\mathrm{B}^4 + n_\mathrm{B}^2 - k_\mathrm{B}^2 + 2n_\mathrm{B}^2 k_\mathrm{B}^2 - 2}{n_\mathrm{B}^4 + k_\mathrm{B}^4 + 4n_\mathrm{B}^2 - 4k_\mathrm{B}^2 + 2n_\mathrm{B}^2 k_\mathrm{B}^2 + 4} + i \frac{6 n_\mathrm{B} k_\mathrm{B}}{n_\mathrm{B}^4 + k_\mathrm{B}^4 + 4n_\mathrm{B}^2 - 4k_\mathrm{B}^2 + 2n_\mathrm{B}^2 k_\mathrm{B}^2 + 4} \right) \quad \text{S12.7}$$

The quantities of interest are the real and imaginary refractive indices of the mixture. We can rearrange the relationship between R_e and m_e described by Eqn. S12.1 to write:

$$m_e = n_e + ik_e = \sqrt{\frac{M_e + 2R_e\rho_e}{M_e - R_e\rho_e}} \quad \text{S12.8}$$

It is not straightforward to write an analytical expression for the real and imaginary roots of the above square root, but nonetheless is straightforward to calculate computationally. Clearly, given that R_e is comprised of real and imaginary components, we write:

$$n_e = \mathrm{Re}\left\{ \sqrt{\frac{M_e + 2R_e\rho_e}{M_e - R_e\rho_e}} \right\} \quad \text{S12.9}$$

$$k_e = \mathrm{Im}\left\{ \sqrt{\frac{M_e + 2R_e\rho_e}{M_e - R_e\rho_e}} \right\} \quad \text{S12.10}$$

Therefore, from calculation of R_e using Eqn. S12.7 in combination with knowledge of n_A, k_A, n_B, k_B, M_A, M_B, ρ_A, and ρ_B for a two component mixture with a known mass fraction x_A, then substituting this equation into Eqn. S12.9 or S12.10 and with knowledge of M_e and ρ_e, the real and imaginary refractive indices of the mixture are predicted. It is important to recognise that this predictive framework of the molar refraction mixing rule applied to light absorbing particles shows that n_e depends on *both* the real *and* imaginary components of the pure components, and similarly for k_e. The predictive treatments for n_e and k_e cannot and strictly must not be separated. We demonstrate this dependence of n_e and k_e on both the real and imaginary components of the pure components by considering a simple two component mixture. The refractive index of pure A is kept constant and takes a value of $m_\mathrm{A} = 1.554 + 0i$ (i.e., the same values for ammonium sulfate at a wavelength of 405 nm). We explore the impact of varying either n_B or k_B; if a given parameter is not varied in a calculation, the constant values of $n_\mathrm{B} = 1.6094$ and $k_\mathrm{B} = 0.1628$ are assumed. The pure component densities of A and B were taken as 1730 kg m^{-3} and 1485 kg m^{-3} respectively, and those for the molecular weight were taken as 132 g mol^{-1} and 645 g mol^{-1} for A and B respectively. The effective molecular weights of the mixtures were treated using Eqn. S12.4, and ρ_e was treated using the ideal mixing rule for mass density (see main manuscript).

The top panels of Figure S14 show the predicted n_e and k_e for different values of k_B, with k_B taking values in the range 0.0 - 0.8 (i.e., spanning a range between a non-absorbing species and a value that represents a strong absorber of visible light such as black carbon) (Bond and Bergstrom 2006). The value of k_B has a clear impact on the real refractive index of the mixture, which would not be predicted by other mixing rules assuming separate treatments of n mixing and k mixing. For example, at a mass fraction of B of 0.5, n_e increases from ~1.58 to 1.66 (i.e., by 5.1%) for k_B increasing from 0.0 to 0.8. Such a discrepancy in n_e arising if one were to ignore the impact of k_B would lead to considerable impacts on the accuracy of extensive aerosol optical properties (e.g., extinction and absorption cross-sections). The bottom panels of S8 show the predicted n_e and k_e for varying k_B over the range 1.4 to 2.0 in 0.1 intervals. The value of n_B impacts significantly on k_e, with k_e decreasing from 0.09 to 0.07 (i.e., by 22%) over the investigated n_B range at a mass fraction of B of 0.5. Again, such large discrepancies

in k_e that would arise from the lack of consideration of the impact of pure component n values in commonly used linear mixing rules could give considerable errors in predictions of extensive aerosol optical properties.

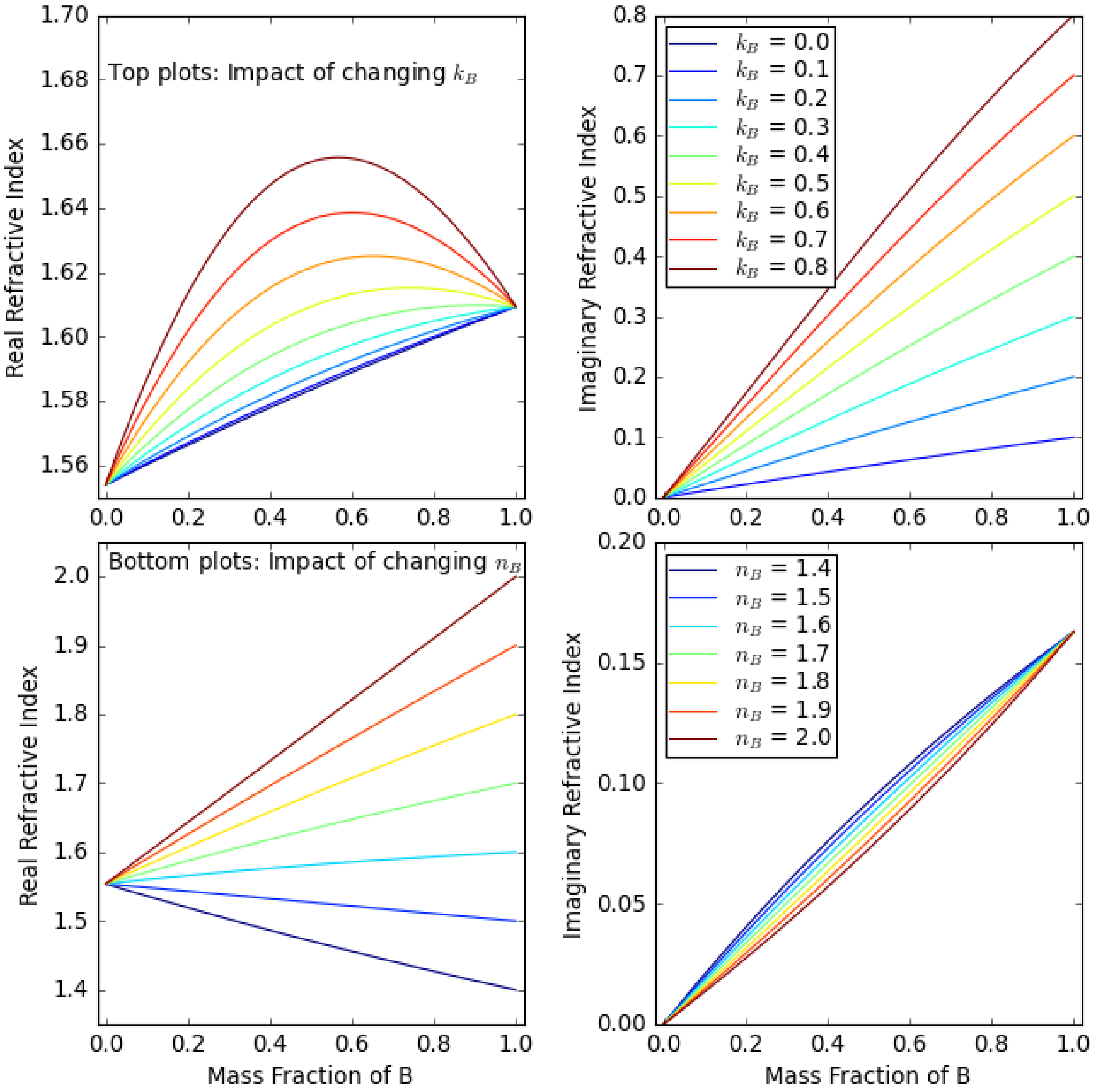

Figure S15. Calculations of effective refractive indices for two-component mixtures of A and B using the molar refraction mixing model. Top panels: The variation of n_e and k_e with mass fraction of B for different values of k_B in the range 0.0-0.8 as indicated in the figure legend. Bottom panels: The variation of n_e and k_e with mass fraction of B for different values of n_B in the range 1.4-2.0 as indicated in the figure legend. The refractive index of pure A is kept constant in all calculations and takes a value of $m_A = 1.554 + 0i$. For B, unless the real or imaginary refractive index parameter is being varied, the constant values of $n_B = 1.6094$ and $k_B = 0.1628$ are used. The pure component densities and molecular weights of A and B, and the effective mass density of the mixture, are treated as described in the main text.

S14. Scanning electron microscopy images of aerosol particles

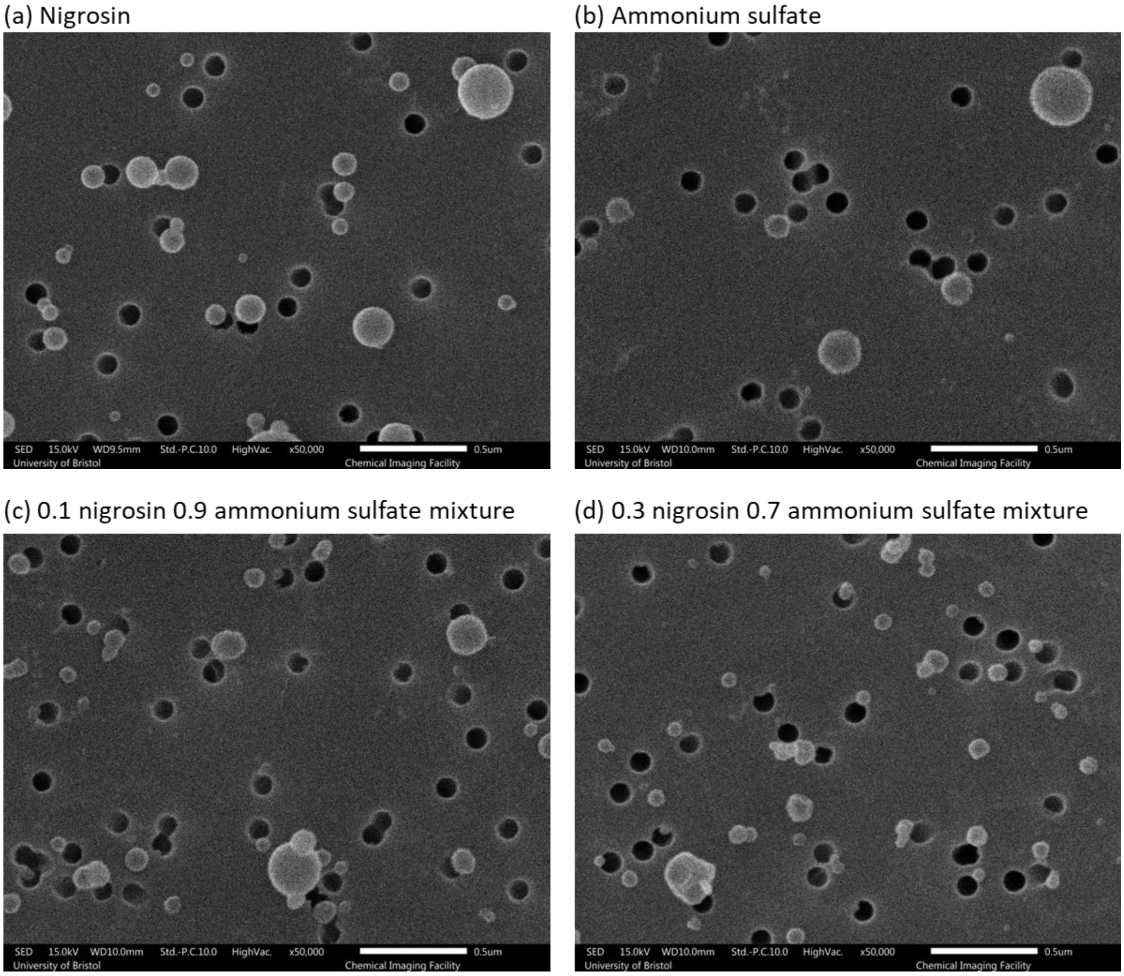

Figure S16. SEM images of aerosol particles before size selection, for: (a) nigrosin; (b) ammonium sulfate; (c) 0.1:0.9 mass ratio of nigrosin:ammonium sulfate; and (d) 0.3:0.7 mass ratio of nigrosin:ammonium sulfate.

References

Bond, T.C. and Bergstrom, R.W. (2006). Light Absorption by Carbonaceous Particles: An Investigative Review. *Aerosol Science and Technology* 40 (1):27–67. doi:10.1080/02786820500421521.

Cotterell, M.I., Szpek, K., Haywood, J.M., and Langridge, J.M. (2020). Sensitivity and accuracy of refractive index retrievals from measured extinction and absorption cross sections for mobility-selected internally mixed light absorbing aerosols. *Aerosol Science and Technology* 54 (9):1034–1057. doi:10.1080/02786826.2020.1757034.

Cotterell, M.I., Ward, G.P., Hibbins, A.P., Wilson, A., Haywood, J.M., and Langridge, J.M. (2019). Optimizing the performance of aerosol photoacoustic cells using a finite element model. Part 2: Application to a two-resonator cell. *Aerosol Science and Technology* 53 (10):1128–1148. doi:10.1080/02786826.2019.1648749.

Davies, N.W., Cotterell, M.I., Fox, C., Szpek, K., Haywood, J.M., and Langridge, J.M. (2018). On the accuracy of aerosol photoacoustic spectrometer calibrations using absorption by ozone. *Atmos Meas Tech* 11 (4):2313–2324. doi:10.5194/AMT-11-2313-2018.

Everest, M.A. and Atkinson, D.B. (2008). Discrete sums for the rapid determination of exponential decay constants. *Review of Scientific Instruments* 79 (2). doi:10.1063/1.2839918.

Liu, Y. and Daum, P.H. (2008). Relationship of refractive index to mass density and self-consistency of mixing rules for multicomponent mixtures like ambient aerosols. *J Aerosol Sci* 39 (11):974–986. doi:10.1016/j.jaerosci.2008.06.006.

Mayerhöfer, T.G. and Popp, J. (2020). Beyond Beer's Law: Revisiting the Lorentz-Lorenz Equation. *ChemPhysChem* 21 (12):1218–1223. doi:10.1002/CPHC.202000301.

Pugh, T.L. and Heuer, W. (1957). Density of polystyrene and polyvinyltoluene latex particles. *J Colloid Sci* 1:173–180. doi:10.1016/0095-8522(57)90004-1.

Stolzenburg, M.R. and McMurry, P.H. (2008). Equations governing single and tandem DMA configurations and a new lognormal approximation to the transfer function. *Aerosol Science and Technology* 42 (6):421–432. doi:10.1080/02786820802157823.

Tabibian, R.M., Heller, W., and Epel, J.N. (1956). Experimental investigations on the light scattering of colloidal spheres I. The specific turbidity. *J Colloid Sci* 11:195–213. doi:10.1016/0095-8522(56)90043-5.

Tigges, L., Jain, A., and Schmid, H.J. (2015a). On the bipolar charge distribution used for mobility particle sizing: Theoretical considerations. *J Aerosol Sci* 88:119–134. doi:10.1016/J.JAEROSCI.2015.05.010.

Tigges, L., Wiedensohler, A., Weinhold, K., Gandhi, J., and Schmid, H.J. (2015b). Bipolar charge distribution of a soft X-ray diffusion charger. *J Aerosol Sci* 90:77–86. doi:10.1016/J.JAEROSCI.2015.07.002.

Williams, R.C. and Backus, R.C. (1949). The Electron-Micrographic Structure of Shadow-Cast Films and Surfaces. *J. Appl. Phys* 20:98–106. doi:10.1063/1.1698244.
